# Counterattacking the tick bite: towards a rational design of anti-tick vaccines targeting pathogen transmission

**DOI:** 10.1186/s13071-019-3468-x

**Published:** 2019-05-14

**Authors:** Ryan O. M. Rego, Jos J. A. Trentelman, Juan Anguita, Ard M. Nijhof, Hein Sprong, Boris Klempa, Ondrej Hajdusek, Julen Tomás-Cortázar, Tal Azagi, Martin Strnad, Sarah Knorr, Radek Sima, Marie Jalovecka, Sabína Fumačová Havlíková, Martina Ličková, Monika Sláviková, Petr Kopacek, Libor Grubhoffer, Joppe W. Hovius

**Affiliations:** 1 Biology Centre, Institute of Parasitology, Czech Academy of Sciences, Branišovská 31, 37005 Ceske Budejovice, Czech Republic; 2 Amsterdam UMC, Location AMC, Center for Experimental and Molecular Medicine, Amsterdam, The Netherlands; 3 CIC bioGUNE, 48160 Derio, Spain; 4 Ikerbasque, Basque Foundation for Science, 48012 Bilbao, Spain; 5 Institute for Parasitology and Tropical Veterinary Medicine, Freie Universität Berlin, Berlin, Germany; 6 Centre for Zoonoses and Environmental Microbiology, Centre for Infectious Disease Control, National Institute for Public Health and the Environment (RIVM), Bilthoven, The Netherlands; 7 Institute of Virology, Biomedical Research Center of the Slovak Academy of Sciences, Bratislava, Slovakia; 8 Faculty of Science, University of South Bohemia, Branišovská 31, 37005 Ceske Budejovice, Czech Republic

**Keywords:** Tick, Vaccine, *Ixodes*, *Borrelia*, TBEV, *Anaplasma*, *Babesia*, *Rickettsia*, Saliva, Midgut

## Abstract

Hematophagous arthropods are responsible for the transmission of a variety of pathogens that cause disease in humans and animals. Ticks of the *Ixodes ricinus* complex are vectors for some of the most frequently occurring human tick-borne diseases, particularly Lyme borreliosis and tick-borne encephalitis virus (TBEV). The search for vaccines against these diseases is ongoing. Efforts during the last few decades have primarily focused on understanding the biology of the transmitted viruses, bacteria and protozoans, with the goal of identifying targets for intervention. Successful vaccines have been developed against TBEV and Lyme borreliosis, although the latter is no longer available for humans. More recently, the focus of intervention has shifted back to where it was initially being studied which is the vector. State of the art technologies are being used for the identification of potential vaccine candidates for anti-tick vaccines that could be used either in humans or animals. The study of the interrelationship between ticks and the pathogens they transmit, including mechanisms of acquisition, persistence and transmission have come to the fore, as this knowledge may lead to the identification of critical elements of the pathogens’ life-cycle that could be targeted by vaccines. Here, we review the status of our current knowledge on the triangular relationships between ticks, the pathogens they carry and the mammalian hosts, as well as methods that are being used to identify anti-tick vaccine candidates that can prevent the transmission of tick-borne pathogens.

## Background

There has been an increasing incidence of several vector-borne diseases, including those that are mosquito-borne, such as Zika and dengue, as well as those that are tick-borne, such as Lyme borreliosis (LB) and tick-borne encephalitis (TBE). In the USA, tick-borne diseases have more than doubled in the last decade, accounting for 77% of all vector-borne diseases, of which 82% of the cases correspond to LB [1]. In Europe, LB is also endemic and considered a public health problem [2]. Indeed, the European Parliament has recently expressed its concerns about the spread of LB in the European population [3]. Besides LB, TBE is endemic in most European countries and has been predicted to increase in the future [4, 5]. Moreover, other less-known tick-borne pathogens (TBPs), including *Borrelia miyamotoi*, *Neoehrlichia mikurensis*, Crimean Congo hemorrhagic fever virus, Powassan virus, Bourbon virus, *Rickettsia* species, *Babesia* species as well as *Anaplasma phagocytophilum* are starting to be slowly recognized as (re)emerging tick-borne diseases [6, 7].

Taken together, TBPs are a major topic on the public health agenda. As with most infectious diseases, a preferred strategy to prevent infection is to identify vaccines targeting individual pathogens. However, because of the variety of microorganisms that ticks are able to transmit, an attractive alternative, and perhaps a more economical approach, would be to target the tick vector itself, either to interfere with tick feeding and/or pathogen transmission [8]. Various strategies for tick control have been experimentally tested, including vaccination or direct acaricide treatment of reservoir hosts, as well as the use of biological methods, such as entomopathogenic fungi to reduce tick populations and/or their colonization by pathogens [9]. However, their success is limited, and these strategies present several drawbacks. For example, the use of acaricides can cause acaricide-resistance, environmental pollution and contamination of dairy and meat products [10, 11]. Therefore, an interesting option that has been gaining traction is the identification of tick antigens that could elicit an immune response in the host and prevent the attachment or feeding of ticks. The idea that an efficient immune response against tick feeding is possible was already described 80 years ago, has been observed in multiple mammalian species since [12] and has been coined as ‘tick immunity’ [13].

However, progress towards the development of an anti-tick vaccine that could mimic natural tick immunity has been slow. This is mainly because we lack complete understanding of the mechanisms that drive the rejection response by tick-immune animals or humans. Work carried out with wood ticks (*Dermacentor andersoni*) and guinea pigs detected high rejection upon a second tick infestation and showed that next to the various immune cell types, including lymphocytes, neutrophils and macrophages, basophils infiltrated in large numbers and degranulated next to the feeding lesion [14]. Basophils are known as a major source of histamine, which was shown to be significantly greater in tick-resistant than in tick-naïve animals. Tick sucking and salivation was reportedly reduced upon histamine release [15] and acquired immunity against ticks has been shown to be lost with ablation of basophils [16], for a different tick species. It has been suggested the reason for the difference in host response to repeated tick challenge is not just the types of immune cells that infiltrate the wound sites, since both, animals that show no rejection and those that do, display relatively similar immune cell profile. The difference appears to be primarily due to differences in the lesion architecture [17]. It may also have been due to how different animals interact at the molecular level in response to immunogenic proteins in tick saliva and their effects on their host tissues [18, 19]. In addition to cellular responses, passive transfer experiments where there is transfer of sera from tick-immune animals to naive animals showed that humoral immune response also plays an important role in tick immunity [20–22].

The quest for tick proteins that can serve as successful recombinant vaccine antigens to induce tick resistance, especially proteins that are conserved across tick species, is based on the above observations. This is where it is important to realize the need for a rational design or plan in the search for an anti-tick vaccine. Antigens, delivery systems, and sometimes adjuvants eliciting predictable immune responses against specific epitopes are involved in such rational designed vaccines [23]. In many cases insufficient knowledge about the mechanisms of protection has hampered successful vaccine development. Therefore, in the recent past, more effort has been devoted to the identification of tick antigens through different approaches. One approach would be to identify antigens in the tick midgut that, when targeted by immune components present in the incoming blood meal, can impact tick feeding success, such as Bm86 [24] or pathogen migration within the vector, as shown with TROSPA [25]. Bm86 is the first and only successful anti-tick vaccine that has been commercialized and is still used today against the tropical cattle tick *Rhipicephalus microplus*. However, *R. microplus* is an entirely different tick species than *I. ricinus*; it only feeds on cattle and has a very short life-cycle that is completed on the animal that the larval stage has infested. The effect of Bm86 vaccination is predominantly the reduction of local tick infestation by interfering in this life-cycle [26–28]. In that light it might be less surprising that vaccination with the *Ixodes ricinus* homologues of Bm86 (Ir86-1 and Ir86-2) did not show any effect on the feeding parameters of *I. ricinus* [29]. Therefore, new antigens are needed for vaccines targeting *Ixodes* ticks. Next to gut proteins, another option that has been favored in the last few years is to identify tick saliva components that may be critical during the feeding process and transmission of one or more pathogens to the mammalian host. These are the main focus of our review. In 2007, Narasimhan and co-workers showed that proteins secreted in the first 24 hours of feeding were sufficient to provide tick immunity in a guinea-pig model which, although feeding ticks were not completely rejected, it drastically blocked *Borrelia* transmission [30]. Which tick proteins elicited this immune response remains to be elucidated. Conserved saliva molecules that could be involved in assisting more than a single pathogen during early dissemination of an infection would make ideal candidate vaccine targets. The identification and elucidation of the function of these proteins formed the foundation of the ANTIDotE project [31].

The important requisites and parameters mentioned above illustrates that anti-tick vaccines could potentially target a broad range of pathogens and tick species. The identification and development of particular antigens as vaccine candidates includes their evaluation, defining their function, their formulation and finally, studies in animal models with infected and uninfected ticks to determine their effectiveness in blocking pathogen transmission and tick feeding. Therefore, strategies to identify anti-tick vaccine antigen(s) should be based on expanding our knowledge of the biology of the tick and its interaction with pathogens. Here, we review work that has focused on pinpointing tick proteins that play a role in the transmission of several *Ixodes*-borne pathogens. We discuss how the identification and functional characterization of selected tick proteins, with a focus on tick salivary gland proteins (Table 1), could help in the fight against the diseases that ticks transmit and discuss their suitability as anti-tick vaccine candidates.

**Table 1:** A selection of tick proteins that have been identified/tested as acquisition or transmission-blocking anti-tick vaccines in *Ixodes* ticks and are discussed in the present review

Tick protein	Tick	Pathogen used for study	Reference
TSLP1	*I. scapularis*, *I. ricinus*	*B. burgdorferi* (*s.l.*)	[50, 51, 53]
tHRF	*I. scapularis*	*B. burgdorferi* (*s.l.*)	[16, 56]
Salp15	*I. scapularis*	*B. burgdorferi* (*s.l.*)	[54, 67]
SUB	*I. scapularis*	*B. burgdorferi* (*s.l.*)	[102]
64P	*I. ricinus*	TBEV	[95, 97]
SUB	*I. ricinus*	TBEV	[104]
SUB	*I. scapularis*	*A. phagocytophilum*	[120, 128]
P11	*I. scapularis*	*A. phagocytophilum*	[119]
Salp16	*I. scapularis*	*A. phagocytophilum*	[118]
IAFGP	*I. scapularis*	*A. phagocytophilum*	[122]
alpha1,3 fucosylytransferase	*I. scapularis*	*A. phagocytophilum*	[123]
lipocalin	*I. ricinus*	*A. phagocytophilum*	[124]
Lectin pathway inhibitor	*I. ricinus*	*A. phagocytophilum*	[124]

## Borrelia

LB is caused by members of the spirochete family from the genus *Borrelia*. These pathogens are able to establish persistent infections both in the tick vector and the vertebrate host. The disease is widely spread in the Northern Hemisphere, albeit with important differences related to the species causing the disease. Thus, while in the USA *B. burgdorferi* (*sensu stricto*) is the dominant genospecies associated with infection, at least two other *B. burgdorferi* (*sensu lato*) (*s.l.*) genospecies, namely *B. afzelii* and *B. garinii* are most commonly responsible for the disease in Eurasia. The number of cases of LB is continuously increasing and suspected to be in the hundreds of thousands both in the USA and Europe. In Europe, there are more than 65,000 documented cases every year [32] with incidences peaking in some countries as high as 350 per 100,000 people. LB is highly prevalent in other continents, such as North America and Asia [33]. In the USA, it is estimated that the number of cases per year surpasses 300,000, which appear particularly in the Northeast, Midwest and the Pacific region [34].

When infected ticks feed on natural or incidental hosts, spirochetes are deposited in the skin. The spirochete has developed tactics to evade killing mechanisms during all stages of the immune response, both innate and acquired, facilitating infection of the host. An early hallmark in most instances of human infection is the appearance of a skin rash (*erythema migrans*) at the inoculation site as a result of local inflammatory responses. The initial skin inflammatory reaction is sometimes accompanied by secondary symptoms such as fever, headache, malaise, myalgia and/or arthralgia. Dissemination of the spirochete results in the colonization of different tissues and/or organs and the appearance of a variety of inflammatory symptoms, including meningoradiculitis, arthritis, and sometimes conduction abnormalities of the heart. Some untreated individuals develop long-lasting forms of the disease, associated with the ongoing infection with the spirochete and late stage disease may include chronic arthritis, chronic neuroborreliosis, or, in Europe, a specific cutaneous lesion named *acrodermatitis chronica atrophicans* [33].

### Tick-*Borrelia* interactions

All arthropod vectors for *B. burgdorferi* (*s.l.*) are ticks belonging to the genus *Ixodes*. *Ixodes pacificus* is the predominant tick species on the West Coast of the USA whereas *I. scapularis* is the dominant tick species on the East Coast [35]. In Europe, *I. ricinus* most frequently bite humans [36, 37], while in Russia and Asia *I. persulcatus* is the main vector for tick-borne diseases [38]. Unlike *B. miyamotoi*, *B. burgdorferi* (*s.l.*) is considered not to be transmitted from female ticks to their offspring, which is called vertical transmission. Remarkably, a small percentage (0.62%) of field-collected larvae were found to be infected with *B. burgdorferi* (*s.l.*) in a recent Dutch study. These larvae were able to transmit *B. burgdorferi* (*s.l.*) to rodents [39]. In contrast, *B. burgdorferi* (*s.l.*) infection rates are on average much higher in nymphal (11.8%) and adult (14.9%) *I. ricinus* ticks [40], corroborating that horizontal transmission, i.e. *via* vertebrate hosts, is the foremost route [41].

Larval ticks acquire *Borrelia* when feeding on an infected host. The spirochete colonizes the midgut aided by the interaction of its outer surface protein A (OspA) to the tick gut protein, tick receptor for OspA (TROSPA) enabling the spirochete to survive the molting process and to persist throughout the tick’s next life-stage [25]. During the next blood meal for the tick, *Borrelia* proliferates and subsequently migrate, upon a series of not completely understood transcriptional changes, from the gut to the salivary glands from where they are secreted into the host [42]. This transition is at least partially, mediated by changes in the interaction of *B. burgdorferi* outer membrane proteins OspA and BBE31 with the tick gut proteins TROSPA and TRE31, respectively [25, 43, 44]. However, for *B. afzelii* migration within *I. ricinus* and transmission is less well-understood and might differ more from *B. burgdorferi* than anticipated [45].

Interestingly, the only human vaccine (LYMErix™) against *Borrelia* that was on the market was based on OspA and targeted the spirochete within the tick. The mechanism of action is that vaccination-induced anti-OspA antibodies enter the tick midgut during feeding and clear the spirochete within the tick [43, 46]. Unfortunately, the vaccine was voluntarily pulled from the market by the manufacturer for multiple reasons, among which claims of alledged side effects [47]. This despite the fact that no long-term adverse effects of vaccination could be observed in a study population of 11,000 subjects [46]. OspA is still being used in veterinary vaccines in Europe and the are ongoing human trials with a modified OspA vaccine [48].

Ticks secrete saliva into the host to facilitate feeding; it contains a variety of proteins that exert immunosuppressive [49], anti-complement [50, 51] or anti-hemostatic [52] functions. As discussed in the introduction, repetitive tick infestations can lead to the development of antibodies against tick salivary gland proteins and are at the base of the tick immunity phenomenon, i.e. hampering the feeding success or even the rejection of ticks by an acquired host immune response [30, 50]. In addition, the interference with host defense mechanisms by some tick salivary gland proteins also appear to facilitate *Borrelia* transmission, e.g. inhibition of the MBL complement cascade by TSLPI [53]. In addition, tick salivary gland proteins, such as Salp15, protect the spirochete directly from host immune responses by binding to proteins present on the surface of the spirochete [54]. Interestingly, *Borrelia* infection can alter the expression of tick proteins, for example the expression of TSLPI, Salp15 and tHRF, providing beneficial effects for the survival of the spirochete in either the tick or the vertebrate host [50, 51, 54–56]. Passive transfer of antibodies from tick immune rabbits or guinea pigs to mice has shown to protect mice against infection when challenged with *B. burgdorferi-*infected *I. scapularis* nymphs [56]. In recent years multiple attempts have been made to identify tick proteins involved in pathogen transmission and indeed, vaccines targeting salivary gland, or midgut, proteins have shown to at least partially reduce *Borrelia* transmission and/or acquisition [25, 53, 54, 56–58]. As there are already excellent reviews published giving a complete overview of all the discovered antigens [59–62], we will discuss here three tick salivary gland proteins in more detail, which have been identified using different approaches and demonstrate the extensive range of tick-host-*Borrelia* interactions that can be targeted.

#### Tick mannose-binding lectin inhibitor (TSLPI)

TSLPI is a glycosylated protein of 8 kD that is expressed in the salivary glands from the feeding tick and subsequently secreted into the host. TSLPI was first discovered in 2011 by probing a yeast surface display expressing the salivary gland transcripts of fully fed *I. scapularis* nymphs with the serum of a tick immune rabbit [50]. More recently, the *I. ricinus* homologue has been identified [51]. TSLPI has been proven to be an interesting candidate for a transmission-blocking anti-tick vaccine as immunization with anti-TSLPI antibodies and knock down of TSLPI expression through RNA interference (RNAi) in ticks result in lower *Borrelia* loads in the skin of mice after *Borrelia-*infected tick challenge [53]. The observed protective effect of TSLPI vaccination on *Borrelia* loads in the skin can be explained by the fact that TSLPI affects the lectin-complement system and can affect complement activation through two mechanisms. First, TSLPI can bind the carbohydrate recognition domains (CRDs) of Mannan-binding lectin (MBL) through its N-glycans and thus inhibit the MBL-lectin pathway [51, 53]. Secondly, TSLPI inhibits the ficolin-lectin pathway by impeding L-FCN binding to Ac-LDL [50, 53]. Inhibition of the complement system at the tick bite site would not only be beneficial for the tick but could also aid the survival of TBPs in the vertebrate host. The latter holds true for *Borrelia*; recombinant TSLPI protects *B. garinii* strain A87S and *B. burgdorferi* strain N40 against complement mediated killing *in vitro* [50, 51, 53]. This effect could be reversed by anti-TSLPI antibodies and might therefore explain the effect of anti-TSLPI antibodies on *Borrelia* loads *in vivo* [36]. Interestingly, the expression of TSLPI is increased in the salivary glands of *B. burgdorferi*-infected ticks compared to naive ticks [53]. *B. burgdorferi* thus influences TSLPI expression in the tick, creating an increased survival chance upon entry into the vertebrate host.

#### Tick histamine release factor (tHRF)

**Fig. 1:**
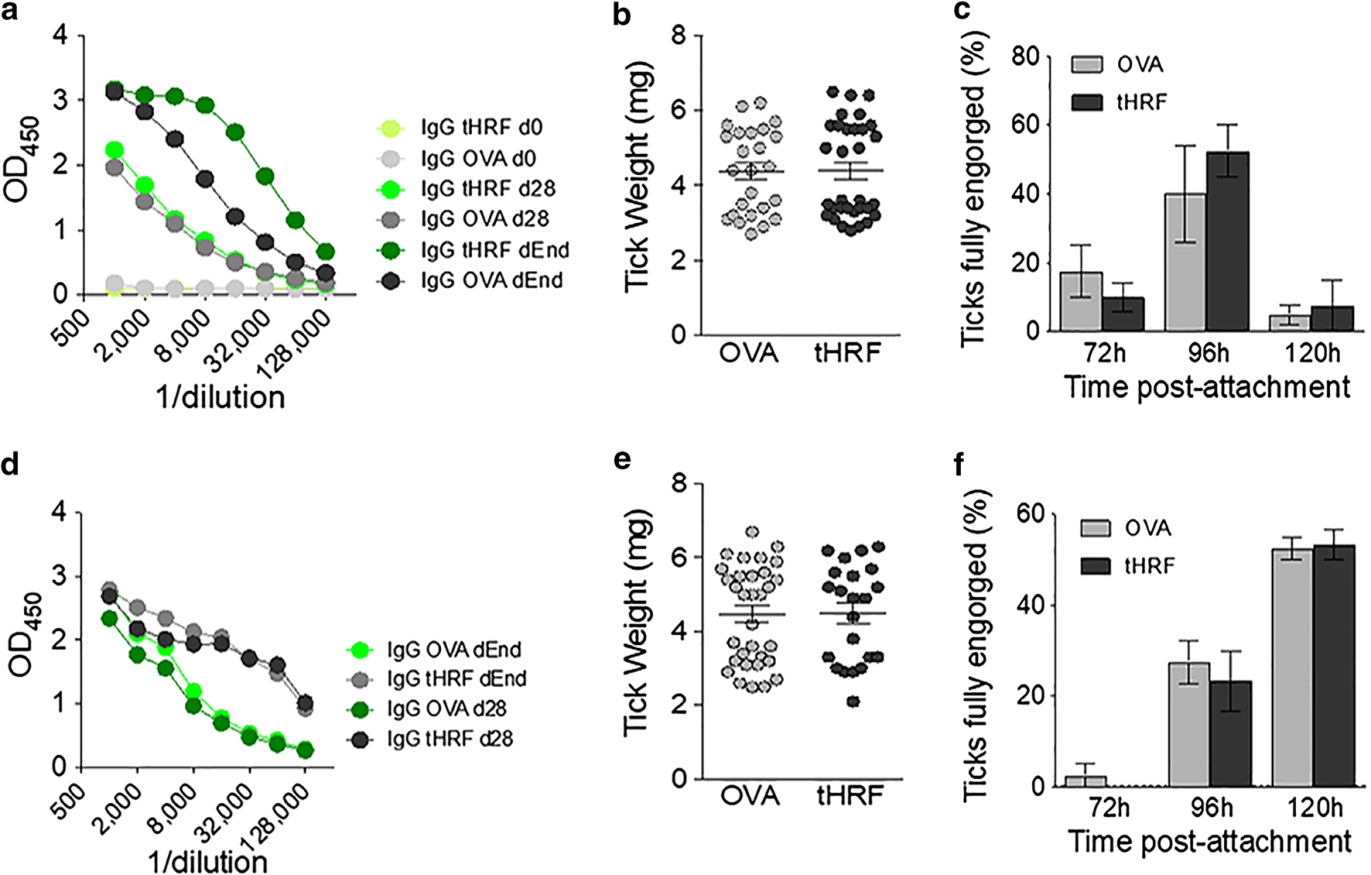
Tick feeding parameters in tHRF immunized mice. **a**, **d** IgG titers in OVA- and tHRF-immunized mice. The sera were tested at the beginning of the assay (IgG d0), after the second boost (IgG d28) and at the end of the experiment (IgG dEnd). The curves represent the mean IgG response from four mice in each group. **b**, **e** Tick weights after feeding on OVA- and tHRF-immunized mice. **c**, **f** Percentage of fully engorged ticks recovered throughout the experiment. The mice were immunized using aluminum hydroxide (**a**–**c**) and Freundʼs adjuvant (**d**–**f**)

tHRF was discovered using 2-dimensional fluorescence difference gel electrophoresis on 66–72 hour fed *B. burgdorferi* infected *I. scapularis* salivary glands [56]. tHRF was found to be upregulated upon *Borrelia* infection and expression levels are highest at 72 hours after attachment. It is a secreted protein, present in both the tick saliva and midgut. Knockdown of tHRF expression in ticks by RNAi resulted in reduced *I. scapularis* nymphal post-engorgement weights, as well as reduced *B. burgdorferi* transmission to the host; *Borrelia* loads were significantly lower in the host skin and deeper tissues [56]. tHRF knock down also significantly reduced *Borrelia* loads in engorged ticks after feeding on a naive host. It was shown that subsequent passive and active vaccination with *E. coli*-produced tHRF, also reduced tick weights and *B. burgdorferi* transmission. Furthermore, vaccination did not only reduce *Borrelia* loads, but 20–33% of the immunized mice were actually found to be PCR-negative for *B. burgdorferi flaB*. Interestingly, tHRF shows homology to the murine histamine release factor (comparison of the protein sequence showed 57.1% similarity and 40.1% identity). Dai et al. [56] showed that tHRF indeed plays a role in histamine release; when recombinant tHRF was incubated with rat basophils *in vitro*, flow cytometry and confocal microscopy showed that tHRF binds to basophils and induces histamine release in a dose-dependent manner. The potency of recombinant tHRF depends on the expression system used, as *E. coli-*produced tHRF induced lower histamine release *in vitro* compared to tHRF produced by *Drosophila* S2 cells. Basophils also appear to be important in anti-tick immunity induced by repeated larval *Haemaphysalis longicornis* infestations, but the mechanism is not completely known [16]. Histamine production could be part of the mechanism as histamine induces itching and promotes the recruitment of pro-inflammatory cells, both important processes in the host response to tick bites [15, 63–65]. Dai et al. also demonstrated that since ticks are sensitive to histamine during the first 24 hours after tick attachment [65], they secrete histamine binding proteins during the early tick feeding phase, but these are reduced in the later feeding phase when tHRF expression increases [56]. It therefore seems that tHRF plays an important role in the late phases of tick feeding when the tick rapidly engorges. The subsequent release of histamine by basophils triggered by tHRF binding, might modulate the vascular permeability and increase the blood flow to the tick bite site, resulting in more blood uptake by the feeding tick. Indeed, injection of recombinant tHRF at the *I. scapularis* bite site 60 hours after tick attachment increased tick weights as well as *B. burgdorferi* loads. Therefore, the effect of anti-tHRF antibodies on histamine release and successive *B. burgdorferi* transmission, might be explained through the reduced tick feeding success as *Borrelia* is triggered by the presence of host blood in the midgut of the tick to proliferate and migrate to the salivary glands [56]. In addition, reduced histamine leads to diminished vascular permeability, which could prevent the successful dissemination of *Borrelia* from the tick-bite site to distal sites. In contrast to *I. scapularis* tHRF, its *I. ricinus* ortholog, which is almost identical except for a conserved modification at amino acid 162 (Val^162^Met), was not able to affect tick feeding in BALB/c mice upon vaccination regardless of the adjuvant used, CFA/IFA or alumn (Fig. 1). These results suggest that there could be significant differences in the immune relationships between mice and *I. scapularis* or *I. ricinus* ticks. Possible explanations include, redundancy in the expression of histamine releasing factor proteins, differential expression of tHRF or the participation of other antigens or immunomodulators of the host immune system.

#### Salivary gland protein of 15 KDa (Salp15)

Salp15 was identified from the salivary glands of *I. scapularis* ticks as one of several antigenic proteins recognized by tick-immune guinea pig antibodies [66] as a 408-bp gene encoding a 14.7 kDa protein, with a signal sequence of 21 amino acids. Vaccination of mice with Salp15 provided protection from infection with *B. burgdorferi* and enhanced the protection provided by the presence of low concentrations of anti-OspA antibodies [67]. Upon further investigation, *in silico* analysis of the amino acid sequence showed weak homology with the active motif region of Inhibin A, a member of the TGF-ß superfamily [68], suggesting that the protein may have immunomodulatory activity. Indeed, Salp15 inhibits the proliferation of CD4 T cells by repressing the production of the autocrine growth factor IL-2. Confocal microscopy localized Salp15 attached to CD4 T cells, but not CD8 T cells. Further studies showed that CD4 is the receptor for Salp15 [69]. Salp15 is able to impede the proper activation of the Src kinase Lck through the induction of a conformational change in CD4 that prevents the binding of the kinase [70]. This results in the inhibition of downstream signaling cascades, and the expression of the *il-2* gene [69, 71]. Besides the inhibition of CD4 T cell activation, Salp15 is able to inhibit dendritic cell function through its interaction with the C-type lectin, DC-SIGN and the activation of the Raf-1/MEK cascade [72]. Moreover, the salivary protein also binds to *B. burgdorferi* OspC (outer surface protein C) and protects complement sensitive bacteria from complement-dependent killing [73], as well as more complement resistant bacteria from antibody-mediated complement-dependent killing, facilitating the survival of the spirochete and thereby pathogen transmission and host infection [54, 67].

The above-mentioned findings might all be involved in the mechanism by which a Salp15-based vaccine exerts its function. It could be that upon vaccination, neutralizing antibodies interfere with the immunosuppressive functions of the protein, thereby making the tick-bite site a more hostile environment for *Borrelia*, as we previously postulated [74]. In addition, it has been shown that the binding of anti-Salp15 antibodies to Salp15-*Borrelia* complexes leads to opsonophagocytosis by phagocytes *in vitro* [67].

There is more beyond the anti-tick vaccine horizon. The effects of Salp15 on CD4 T cells have also made this saliva protein an interesting candidate as a therapeutic agent in pathologies mediated by these cells. In a mouse model of allergic asthma Salp15 prevented the development of ovalbumin-induced pathology [75]; however, the tick saliva protein does not seem to affect memory or effector CD4 T cells. The therapeutic effect of Salp15 has also been tested in other models of immune pathology, such as experimental autoimmune encephalomyelitis (EAE), a mouse model for multiple sclerosis [76] or graft versus host disease [52]. These studies underscore that the identification of tick antigens and unravelling their functions can have much broader implications than merely expanding the arsenal of anti-tick vaccine candidates.

## Tick-borne encephalitis virus (TBEV)

TBEV is medically the most important tick-borne virus in Eurasia. TBE is considered to be a growing public health concern not only due to an increase of the incidence in some risk areas, but also due to expansion of risk areas and the identification of new natural foci [77, 78]. The incidence of TBE is reported to be between 10,000 and 15,000 cases per year worldwide, but it is considered to be underestimated [5]. The highest incidences of clinical cases are recently reported from the Baltic States, Slovenia and the Russian Federation [79, 80].

The most common route of TBEV infection of humans is through the bite of an infected tick. Less frequently, the infection can be transmitted by the alimentary route involving non-pasteurized dairy products from acutely infected livestock (goats, sheep, cattle) [77]. The clinical manifestations caused by TBEV range from asymptomatic infections and fevers with complete recovery of patients, to debilitating or even fatal encephalitis [81]. The development of clinical manifestations of varying severity seems to be associated with the three virus subtypes. Far Eastern TBEV-subtype is considered to be the most virulent pathogen with a 20–40% case fatality rate and the most severe form of central nervous system disorder. The Siberian TBEV-subtype characteristically induces a less severe acute disease (case fatality rate 6–8%), but with a tendency for patients to develop a chronic form of TBE. The disease caused by European subtype is biphasic with fever during the first phase and neurological disorders of differing severity, during the second phase. The infections are usually milder and more often without serious sequelae [81].

Human TBEV infection can be prevented by vaccines targeting the virus directly. There are currently four vaccines available in Europe and Russia, which are produced according to the WHO manufacturing requirements. These vaccines are reported to be safe and highly immunogenic with a field effectiveness of up to 99% (reviewed in [82] and [80]). Despite the high effectiveness and safety, vaccination coverage in many highly endemic countries is low. This clearly indicates the need for improvement of TBEV vaccination coverage. Universal transmission-blocking anti-tick vaccines providing protection against *Borrelia* sp., *Babesia* sp. and other tick-borne pathogens could serve as an attractive additional or alternative measure with higher vaccination coverage than the standard vaccines and could thereby improve the overall level of protection against TBEV.

In nature, TBEV is maintained in a cycle involving ticks and wild vertebrate hosts, particularly small rodents. The principal vectors of TBEV are *I. ricinus* (associated with the European TBEV subtype) and *I. persulcatus* (associated with the Siberian and Far-Eastern TBEV subtypes) ticks. Several mechanisms of virus transmission in nature are described. Vertical transmission of the virus in the form of transovarial transmission of the virions *via* the eggs, as well as transstadial transmission has been documented. Transstadial transmission seems to be ineffective and its importance to the maintenance of the virus in nature is considered to be rather low [83–85]. Horizontal means of virus transmission play a crucial role in the maintenance of tick-borne viruses in nature, where viraemic animals can serve as a virus source for the feeding ticks. The virus replicates in the tick, which transmit it to a naive vertebrate host when they take a second blood meal. This so called viraemic transmission was for decades considered to be the main route of TBEV circulation in nature. However, another important mechanism of virus circulation in nature is non-viraemic transmission of the virus from infected to non-infected ticks when they co-feed on the same host [86]. The co-feeding ticks become infected also when the hosts have very low or undetectable viraemia and even in the presence of TBEV-neutralizing antibodies [87]. The local skin site where ticks feed has been shown to be an important focus of viral replication where migratory immune cells provide a vehicle for virus transmission from infected to uninfected co-feeding ticks [88]. The virus transmission is indirectly promoted *via* the actions of tick saliva molecules in the vertebrate host, a phenomenon designated “saliva-assisted transmission” (SAT; [89]). As discussed in more detail elsewhere in this review, ticks succeed in feeding by injecting a cocktail of salivary molecules into the feeding pool with a broad spectrum of antihaemostatic and immunomodulatory functions such as inhibitors of the pain and itch response, anticoagulants, antiplatelet components, vasodilators, and immunomodulators (recently reviewed in [90] and [91]). The molecules involved in SAT can be considered as the most promising targets for developing a transmission blocking anti-tick vaccine.

However, only limited data are available on SAT molecules in the context of TBEV or viral infections in general. One of the examples of SAT factors is sialostatin L2, inhibitor of cysteine peptidases, which have been characterized in the tick *I. scapularis* [49, 92]. Lieskovská et al. [93] recently reported that sialostatin L2 attenuates the interferon β mediated immune reactions in mouse dendritic cells. Consequently, the suppression of interferon-stimulated genes led to the enhancement of the TBEV replication in dendritic cells. This might be a mechanism by which tick saliva facilitates virus transmission and thereby increases the virus transmission efficiency.

Surprisingly, only few anti-tick vaccine candidates have been studied directly in the context of tick-borne viruses. Recently, the vaccine potential of *Hyalomma anatolicum* ticks-derived molecules ferritin 2 and tropomyosin has been studied with the aim to develop anti-Hyalomma vaccine which could help to reduce infections of Crimean-Congo haemorrhagic fever (CCHF) virus in the domestic animals. Both vaccine candidates showed partial protection of immunized cross-bred male calves against challenge tick infestations (51.2–66.4%). However, their direct effect on virus transmission by a challenge with CCHFV-infected ticks has not been assessed [94]. So far, only two candidates have been evaluated for their direct effect on virus transmission, both in connection with TBEV. The first candidate is the tick protein 64P, a 15-kDa cement protein of the tick *Rhipicephalus appendiculatus* [95]. The protein is derived from the cement cone that anchors the tick’s mouthparts in the host skin, but antibodies against 64P were also found to cross-react with antigenic epitopes in the tick midgut. To expose immunoprotective regions within 64P, four truncated versions of the protein and two full-length clones (64TRP1-6) were expressed in *E. coli* using a GST/HIS.TAG-fusion protein expression system [96]. Recombinant forms of 64P (64TRP) were effective against adult and immature stages of several tick species, including *I. ricinus* and induced potent humoral and delayed type hypersensitivity responses. In hamster, guinea pig, and rabbit models, this cement antigen acts as a dual-action vaccine by targeting the tick-feeding site (impairing attachment and feeding) and cross-reacting with the “concealed” midgut antigens, resulting in the death of engorged ticks [95, 96]. Labuda et al. [97] tested the potential of the 64TRP anti-tick vaccine to protect mice against a lethal infection of TBEV transmitted by its natural vector, *I. ricinus* [97]. Transmission-blocking and protective activities were demonstrated by the 64TRP vaccine. The highest level of protection from a single 64TRP dose was observed with TRP6 (71% survival). This construct shows the most extensive antigenic cross-reactivity with whole nymphal extracts, cement cone, and midgut of female *I. ricinus* [95]. 64TRP-immunized mice developed antiviral protection even when they did not support virus transmission to co-feeding nymphs. These data indicate that the response of 64TRP-immunized mice to tick feeding did not completely block virus transmission, but instead allowed sufficient exposure to the virus for the mouse to develop protective immunity. The protective effect of immunization with a single dose of the 64TRP tick antigens did not differ significantly from a single shot of the commercially available inactivated TBEV vaccine (FSME-IMMUN; Baxter, Vienna, Austria). Immunization with the commercial TickGARD vaccine blocked transmission similar to 64TRP-immunization. However, unlike 64TRP-immunization, the transmission-blocking effects of TickGARD did not provide protection against lethal infection with TBEV. In conclusion, the 64TRP vaccine demonstrates the potential for a transmission-blocking vaccine, most likely by mediating a local cutaneous inflammatory immune response (delayed type hypersensitivity response) at the tick-feeding site [97].

The second anti-tick vaccine candidate studied in the context of TBEV infection is subolesin (SUB). SUB is a conserved tick protective antigen which is involved in tick innate immunity [98]. It is the ortholog of insect akirin [99, 100]. SUB participates in tick molecular pathways involved in feeding, fertility, pathogen infection and multiplication in ticks [98, 101]. SUB-immunization has been shown to protect against tick infestations and infection by different vector-borne pathogens, e.g. immunization with recombinant SUB showed a reduction of tick infestations and transmission of *A. phagocytophilum*, *A. marginale*, *Babesia bigemina* and *B. burgdorferi* [102, 103]. Havlíková et al. [104] studied the effect of SUB and SUB-imunization on TBEV infection in ticks, transmission of the virus during feeding and course of infection in immunized mice. Results showed that SUB expression is downregulated during the *I. ricinus* tick feeding. However, TBEV infection increases SUB mRNA levels in tick tissues, thus supporting a role for this molecule in tick innate response to virus infection. Immunization with recombinant SUB reduced SUB mRNA levels in nymphs co-feeding with infected females. However, the vaccination with SUB not only failed to protect the mice from TBE-induced encephalitis, but rather led to slightly increased virus titers in infected female ticks and co-feeding nymphs, which obtained the virus through non-viraemic transmission. The example of SUB illustrates the complexity of tick innate immunity and its interplay with the factors involved in SAT of various TBPs. It also highlights the necessity to always take tick-borne viral infections into consideration in the efforts to develop anti-tick vaccines even if blocking of virus transmission is not necessarily the primary goal of the anti-tick vaccine development efforts.

## Anaplasma phagocytophilum

*Anaplasma phagocytophilum* is an intracellular bacterium and causal agent of human granulocytic anaplasmosis (HGA), equine and canine granulocytic anaplasmosis and tick-borne fever (TBF) in ruminants [105]. Although the veterinary relevance of *A. phagocytophilum* has been known for decades [106], its zoonotic potential was only recognized in the 1990s [107]. It is an emerging TBP in the northern hemisphere in areas where vector ticks of the *I. persulcatus* complex (*I. persulcatus* in Asia, *I. pacificus* and *I. scapularis* in North America and *I. ricinus* in Europe) are present [108]. In humans, an infection with *A. phagocytophilum* is associated with a nonspecific febrile illness; the clinical presentation of HGA ranges from asymptomatic infections to a sometimes fatal disease [109]. The pathogen infects and propagates primarily in the neutrophils of the vertebrate host, where it survives by manipulating the cellular immune response and inhibiting apoptosis (reviewed in [110]). When taken up with an infected blood meal, *A. phagocytophilum* initially infects tick midgut cells from where it migrates to secretory acini of the salivary glands. It can be transmitted to the next host after the tick molts to the next life-stage and takes up a new blood meal [111].

A vaccine for any of the diseases associated with *A. phagocytophilum* infection is currently not available. Efforts to protect lambs against TBF by common vaccination strategies, such as the use of inactivated *A. phagocytophilum* as antigen, failed [112]. Other strategies have focused on the identification of *A. phagocytophilum* proteins involved in the infection of vertebrate host cells or cell surface proteins as potential vaccine targets. This includes the identification of three *A. phagocytophilum* invasins: outer membrane protein A (ompA), a 14-kDa surface protein (Asp14) and an invasion protein A (AipA) [113–115]. The incubation of *A. phagocytophilum* dense core (DC) organisms with antisera raised against these invasins reduced *A. phagocytophilum* cell entry of mammalian host cells. This blocking effect which was shown to be synergistic as the most effective blocking was observed when dense core organisms were incubated with antibodies against all three invasins [115, 116].

As outlined in the introduction, knowledge on the molecular details of tick-pathogen interactions might lead to the development of novel strategies aimed at interrupting the pathogen transmission cycle. On the other hand, immunization with proteins shown to play a role in tick-host-pathogen interactions does not necessarily result in full protection against subsequent challenge (e.g. [117]). This may be caused by several factors, including genetic diversity, limited antigen immunogenicity or the existence of alternative mechanisms of infection. An increasing number of tick proteins have been identified that play a role in *A. phagocytophilum*-tick interactions using methods such as quantitative transcriptomics, proteomics and metabolomics [118–121]. This includes P11, Salp16, an antifreeze glycoprotein (IAFGP) and alpha1-3-fucosyltransferase [118, 119, 122–124]. P11 is a ~ 11.8 kDa protein expressed in both the salivary glands and haemocytes of *I. scapularis.* Its expression is induced upon *A. phagocytophilum* infection and the protein was shown to bind to *A. phagocytophilum* and facilitate the uptake of the pathogen by tick haemocytes, suggesting that haemocytes ferry the pathogen from the midgut cells to the salivary gland acini. An experiment in which the haemocoel of ticks was injected with P11 antibodies, followed by feeding of these ticks on *A. phagocytophilum*-infected mice resulted in a reduced pathogen burden in the salivary glands and haemolymph, but not in the midgut. Passive immunization of *A. phagocytophilum*-infected mice with rabbit anti-P11 serum, followed by feeding of naïve nymphs on the immunized mice gave similar results [119].

Infection of ticks by *A. phagocytophilum* also promotes the expression of tick salivary gland protein Salp16 through actin phosphorylation, a process dependent on *Ixodes* p21-activated kinase (IPAK1)-mediated signaling [125]. Salp16 was shown to be essential for *A. phagocytophilum* colonization of the tick salivary glands, as demonstrated by RNAi studies [118]. Remarkably, IAFGP, critical for the survival of ticks at cold temperatures, was also found to be more abundantly expressed in ticks following infection with *A. phagocytophilum* [122]. This is suggestive of a mutualistic effect of the pathogen on its tick vector. IAFGP was also shown to inhibit the formation of bacterial biofilms, thereby altering the microbiota in the tick gut and enhancing colonization of the tick by *A. phagocytophilum* [126, 127]. Another gene upregulated upon *A. phagocytophilum* infection is that of alpha 1-3-fucosyltransferease. RNAi-mediated gene silencing of alpha 1-3-fucosyltransferase reduced the capacity of *A. phagocytophilum* to infect tick salivary gland cells [123].

Two other proteins, a salivary lipocalin and a secreted lectin pathway inhibitor were also found to be upregulated upon *A. phagocytophilum* infection in ticks [124]. Both proteins are thought to be involved in the evasion of the host immune response by reducing host inflammatory responses and by inhibiting the complement lectin pathway, respectively. When IgG antibodies raised against these proteins were fed, using an artificial feeding system, to *I. ricinus* ticks, a slight decrease in tick feeding success and fecundity was observed in the group that had fed on anti-lectin pathway inhibitor antibodies [124]. The only other tick antigen known to be upregulated upon *A. phagocytophilum* infection for which active immunization studies have been reported is SUB [120, 128], which has also been discussed in the TBEV section of this review. The feeding of *Ixodes* ticks on SUB-immunized animals was hampered (reviewed in [129]) and nymphs fed as larvae on *A. phagocytophilum*-infected mice immunized with recombinant SUB had reduced pathogen levels [128]. These findings demonstrate that more knowledge about tick-host-pathogen interactions in HGA and TBF is needed to identify candidates for anti-tick vaccines that could interfere with *A. phagocytophilum* transmission from the tick to the host.

## Rickettsia

Rickettsia are gram negative obligate intracellular bacteria that are transmitted to humans through various vectors [130, 131]. Several rickettsial species are pathogenic, and in Europe, species belonging to the Spotted Fever Group (SFG) Rickettsiae, such as *R. massiliae*, *R. conorii*, *R. slovaca*, *R. raoultii*, *R. sibirica*, *R. mongolotimonae*, *R. helvetica* and *R. monacensis* are transmitted by ticks [130, 131]. Although under continuous investigation, there is no available vaccine against rickettsioses [132]. Unlike flea-borne typhus-group *Rickettsia* that may spread quickly among humans, for tick-borne SFG rickettsiae, humans appear to be accidental, and probably dead-end, hosts [132]. Moreover, SFG rickettsioses in Europe are usually well managed with antibiotics [132, 133], which could raise questions regarding the necessity for a dedicated vaccine and hence favor preventative strategies based on anti-tick vaccines targeting transmission of multiple TBPs.

As obligate intracellular bacteria, SFG rickettsiae are required to invade their host’s cells, thus they have evolved various specific processes [134] that could in principle be disrupted to interfere with their infectivity. Many efforts to achieve effective and long-lasting immunity against the highly pathogenic *R. rickettsii* have been undertaken using sub-unit or whole killed bacteria [135]. Unfortunately, inactivated *R. rickettsii-*based vaccines provided only limited immunity by shortening the course of illness or by reducing case fatality rates [135, 136]. Subunit vaccines based on outer membrane proteins were developed for both *R. rickettsii* and *R. conorii* but did not result in long-lasting immunity [135, 136]. Thus, classical approaches to develop a vaccine against SFG rickettsiae have not been successful so far. An anti-tick vaccine based on the interaction between the SFG rickettsiae and the tick might provide an alternative approach, particularly when effective against multiple tick-borne diseases.

Through molecular and biochemical inhibition assays several potential candidates for the disruption of tick cell invasion and pathogen transmission have come to light [137]. SFG rickettsiae appear to interact with their host’s cells actin machinery, be it arthropod or mammal, in order to spread between cells through actin-based motility (ABM) [134, 138, 139]. This phenomenon has been described in detail for other intracellular bacteria such as *Listeria monocytogenes*, which interacts with the hosts cell machinery in order to induce the polymerization of actin filaments, thus providing *L. monocytogenes* cytoplasmic motility [139, 140]. Although most pathogens spreading through ABM use the same pre-existing host pathways, they appear to interact with it in different manners [141]. More knowledge on these specific interactions could perhaps be used in order to interfere with cell to cell spread of specific bacteria. The protein complex Arp2/3 is a major component in the regulation of the actin cytoskeleton of most eukaryotic cells [142] and various studies - both in mammalian and tick cell lines - have shown this complex is recruited by SFG *Rickettsia* in order to enter their host’s cells through endocytosis [134, 142–144]. Studies using varying concentrations of an Arp2/3 complex inhibitor and transcriptional profiles of infected versus uninfected *Dermacentor variabilis* cells established its importance for *R. montanensis* invasion [142, 144]. Similar results have been shown for *R. monacensis*, *R. conorii* and *R. rickettsii* by examining rickettsial proteins that interact with the Arp2/3 complex, such as RickA [143, 145, 146]. Other host proteins involved in rickettsial cell invasion, such as Cdc42, PI 3-kinases, phosphotyrosine kinase (PTK), c-Src, focal adhesion kinase (FAK), Ku70, V-ATPase, α-catenin, Rho GTPases Rac1 and N-WASP have also been investigated to a lesser extent [134, 142, 143, 147, 148]. However, differences were observed between *Rickettsia* species. For example, Rho GTPases Rac1 were found to play an important role in the internalization of *R. montanensis* into *D. variabilis* cells, while they were found unnecessary for *R. conorii* invasion of VERO cells [142, 143]. These results could be accredited to differences in the rickettsial-host interaction between rickettsial species. However, they could also be related to the difference in methods (biochemical inhibition and signaling disruption respectively) or due to the use of arthropod *versus* mammalian cells.

Crossing of the midgut barrier and colonization of tick salivary glands are imperative processes for pathogen transmission *via* tick saliva [6]. A study utilizing both differential-display and subtractive-hybridization PCR in *R. montanensis*-infected *D. variabilis* females found differential expression of nine clones with homology to known proteins, including a putative salivary gland protein SGS-3 precursor (Oi312-SGS-3), which was significantly downregulated in the salivary glands of infected females, while tubulin α-chain (Oi1013-tubulin α-chain) and Ena/vasodilator-stimulated phosphoprotein-like protein (Oi619-VASP) were upregulated. Also, they found that these three, as well as six more putative proteins [vascular-proton-translocating ATPase A isoform 1/clathrin-coated vesicle (Oi6113-clathrin-coated V-ATPase), peroxisomal farnesylated protein (Oi411-PfX), α-catenin, cadherin (Oi812-α-catenin), copper-transporting ATPase (Oi212-Cu2+ ATPase), glycine-rich protein (Oi814-GRP), and Dreg-2 protein (Oi616-Dreg-2)] were downregulated in the tick midgut. The proteins identified in this study might be involved in cell invasion and the host’s stress response [149]. Interestingly, in a later study examining differential expression of putative immune-like tick-derived factors in *D. variabilis* when infected by *R. montanensis* or *R. amblyommii*, it was found that rickettsial exposure downregulated the expression of S-transferase 1 (dvgst1) and Kunitz protease inhibitor (dvkpi) in the midgut. This suggests that rickettsial infection of the midgut might involve the downregulation of the tick’s immune molecules [150]. The tick immune and stress response to rickettsial infection have been evaluated in other studies, finding proteins such as α-2 macroglobulin and ferritin which are involved in the inhibition of exo-proteases of parasites and the reduction of cell damage respectively [151, 152].

Despite the well-described abundance of data on proteins involved in the mediation of rickettsial infection in ticks and their subsequent transmission, it is difficult to predict which proteins are most suitable as targets for transmission-blocking vaccines and experimental evidence using immunized hosts is lacking. Moreover, different *Rickettsia* species were shown to elicit a different response in their tick host [142, 143] and there is much to be learned about the interaction between *R. helvetica* and *R. monacensis* with *I. ricinus* ticks. *Rickettsia helvetica* has been associated with disease, but the extent of its pathogenicity is still being studied and under debate [153, 154]. Cell invasion by *R. monacensis* appears to be similar to that of other pathogenic SFG rickettsiae [146, 155]. In contrast, *R. helvetica* showed disrupted or truncated amino acid sequences in genes encoding proteins involved in cell invasion in SFG *Rickettsia* and confocal laser scanning microscopy revealed the bacteria spread by cell breakdown rather than cell to cell spread [154]. This could mean that antigens targeting proteins found in the studies described above might not be useful for the disruption of *R. helvetica* colonization and transmission by the tick host. In light of the apparent similarities between *R. helvetica* and non-pathogenic *Rickettsia* species, its high prevalence in tick populations and effective vertical transmission [156], its effect on tick fitness should be evaluated. In the last couple of decades, a plethora of information regarding the relationship between arthropods and their endosymbionts has surfaced, becoming more intricate with the use of new high throughput technologies that allow for the analysis of microbiomes [157, 158]. Mutualistic tick-endosymbiont relationships have been described for *Coxiella*, *Francisella* and *Rickettsia*, and have been shown to affect tick fertility, overall fitness and possibly even vectorial capacity [157, 159]. If such effects were to be found between *I. ricinus* ticks and *R. helvetica*, interference of the underlying processes involved could be exploited in order to affect *I. ricinus* fitness and/or pathogen transmission. These findings further highlight the importance of the examination of the specific mechanisms involved in *R. monacensis-I. ricinus* and *R. helvetica-I. ricinus* interactions.

## Babesia

*Babesia* species, the causative agents of babesiosis, are apicomplexan malaria-like parasites of the red blood cells transmitted by *Ixodes* ticks. They are referred to as piroplasms, together with *Theileria* and *Cytauxzoon* species, because of their pear-shaped intra-erythrocytic stage. *Babesia* species infect a wide spectrum of mammalian hosts as well as several avian species and are, after trypanosomes, the most common group of blood parasites [160]. Babesiosis is one of the most common blood diseases of free-living animals [160, 161] and is considered as an emerging zoonosis of humans [160–164]. From a veterinary point of view, most attention is paid to bovine babesiosis, which is responsible for large economic losses to the livestock industry [165]. Bovine babesiosis, is associated with mortalities, abortions, decreased meat as well as milk production and the majority of the world’s cattle population is exposed to the causal agents of babesiosis [165, 166]. In tropical and subtropical areas of Australia, Africa, Asia and the Americas, *Babesia bovis* and *Babesia bigemina* are transmitted by *Rhipicephalus* spp. ticks. In Europe, the disease is mainly caused by *Babesia divergens* and transmitted by *I. ricinus* (reviewed in [162]). Equine piroplasmosis, a disease of horses and donkeys caused by *B. caballi* and *Theileria equi*, and canine babesiosis caused by *B. canis*, *B. rossi*, *B. gibsoni* or *B. vogeli* are examples of other diseases of veterinary relevance that have been reported from many countries (reviewed in [167, 168]).

The current protection against bovine babesiosis is based mostly on the vaccination of young cattle with live attenuated parasites. The animals inoculated with *Babesia*-infected blood show less severe symptoms than naturally-infected animals and develop a protective immunity upon recovery (reviewed in [169]). Recently, the ability of genetic manipulations of the parasite opens ways for production of more efficient, stable, and safe parasite vaccines [170]. Moreover, sequencing of several *Babesia* genomes deepens knowledge about the parasite and its interaction with the host [169]. The *Babesia* antigens, like apical membrane antigen (AMA) [171–176], thrombospondin-related anonymous protein (TRAP) [177, 178] rhoptry-associated protein (RAP) [167, 179–181], merozoite surface antigen (MSA) [164–167], P0 proteins, spherical body proteins (SBP), VESA1 [182], subtilisin-like protein (SUB) [183], and GPI-anchored proteins [184] are potential targets for the vaccine. Last but not least, *Babesia* exoantigens, proteins released in the medium during parasite cultivation, have immunological capacities to reduce severity of the infection, as shown for the current commercialized vaccine against canine babesiosis [185].

Humans are not natural, but accidental hosts for *Babesia* (reviewed in [186]). Nevertheless, clinical cases of human babesiosis have been reported from many countries all over the world (reviewed in [186, 187]). In Europe, infections with *B. divergens*, the main causative agent of human babesiosis, has led to more than 40 medical cases to date [171, 187, 188]. Cases of human babesiosis have also been reported in Africa, Asia, Australia and South America (reviewed in [171, 177, 186, 187]). Currently there is no babesiosis vaccine for humans. Babesiosis can be mistaken for malaria due to mimicry of somatic symptoms in the acute phase but lacks the typical periodicity. Most immunocompetent individuals suffer from flu-like symptoms and recover completely from babesiosis (reviewed in [160, 177, 179]). A more severe infection and disease generally occurs in people with immunosuppressive medication [172, 180], in malignancy [180], after splenectomy [180, 182, 183] or with HIV infection [189–191]. Interestingly, more severe symptoms also occur in patients co-infected with *B. burgdorferi* (*s.l.*) [192, 193].

In Europe, the transmission of species of medical relevance is caused by *I. ricinus* [194]. These ticks serve as the main vector of *B. divergens* and have recently also been identified as the primary vector of *B. venatorum* (also reported as *Babesia* sp. EU1) [195–199]. In addition, *I. ricinus* has been identified as a competent vector of *B. capreoli* [200] and *B. microti* [201].

*Babesia* parasites multiply asexually in the erythrocytes of the vertebrate host where the first sexual stages, gametocytes, occur [202, 203]. The sexual reproduction then occurs in the gut lumen of the tick vector, which starts with maturation of ingested gametocytes and production of gametes. During the next blood-feeding, sporogony takes place in the tick salivary glands and fully matured sporozoites released in the tick saliva invade host erythrocytes *via* the tick bite [204, 205].

An infection with *Babesia* parasites negatively affects tick development [206], so the ticks are thought to have evolved specific immune mechanisms that could limit the *Babesia* infection to tolerable levels [207]. Longicin, a defensin-like protein with anti-microbial and anti-fungal activities, inhibited proliferation of *T. equi* in *in vitro* cultures, reduced the parasitaemia in mice infected with *B. microti* and was shown to play a role in regulating the vectorial capacity of the tick for *Babesia* [208, 209]. Similarly, a recombinant version of a tick midgut cysteine protease named longipain also inhibited proliferation of *T. equi in vitro* and silencing of this gene in ticks by RNAi increased infection of the tick organs [210]. Cystatin-2, a cysteine protease inhibitor, is overexpressed in the tick after *Babesia* infection and the recombinant protein affected the growth of *B. bovis* in *in vitro* cultures [211]. Silencing of vitellogenin receptor, a receptor responsible for the uptake of vitellogenin into the eggs, prevented infection of tick ovaries by *B. gibsoni* [212]. Although several transcriptomic projects identified tick genes upregulated upon presence of the parasite [213–216], up to date only few tick genes have been shown to be directly involved in the parasite acquisition. Silencing of the identified genes, namely TROSPA, serum amyloid A, calreticulin [213], and SUB [103] by RNAi, reduced parasite acquisition by the tick. To our knowledge, no tick proteins that facilitate *Babesia* transmission from the tick to the host have been identified or investigated, let alone investigated as candidates for anti-tick vaccines interfering with *Babesia* transmission.

## Future directions

### Tick-host-pathogen interactions

This review aims at highlighting the efforts in pursuing tick proteins that are responsible for pathogen transmission and hence could serve as candidates for anti-tick vaccines. For *Borrelia* and TBEV, multiple relevant studies have been conducted. Indeed, multiple tick proteins assist *Borrelia* with survival in the tick, transmission from the tick and subsequent successful infection of the vertebrate host. This is either through direct binding to the spirochete or by interacting with host factors to create favorable conditions for *Borrelia* survival. For TBEV, direct interactions of the virus with tick proteins has not been shown. However, there is experimental evidence that the tick protein sialostatin L2 increases TBEV survival by interacting with host factors (dendritic cells). In addition, immune responses to other tick proteins affect TBEV transmission from the tick to the host. Tick-host-pathogen interactions for other TBPs are less well described. For the obligate intracellular bacteria *A. phagocytophilum* and SFG-*Rickettsia* the mechanisms for cell-invasion and cell-to-cell spread are being investigated, and several bacterial proteins involved in these processes have been described. Whether these bacteria apply the same mechanisms and interact with similar host proteins in tick and host cells, remains to be elucidated. Experimental work has also shown that the presence of TBPs can be beneficial for the tick. For example, *A*. *phagocytophilum* induces ticks to express an antifreeze glycoprotein gene that enhances their survival in the cold [122]. Conversely, the tick immune system suppresses the presence/abundance of other TBPs, such as *Babesia*. Studies focusing on the transcriptome or proteome of both the tick and the TBP during acquisition and transmission might help us to determine the key proteins involved in the pathogen-tick interactions.

We have reviewed several tick proteins that have proven to affect transmission of various pathogens from the tick to the host. Unfortunately, their use as potential transmission-blocking vaccines has met limited success when tested as single vaccine formulations. One explanation could be the enormous evolutionary pressure on these proteins (and the encoding genes) as they are readily exposed to the immune system of multiple hosts and to a wide range of pathogens. Indeed, Van Zee et al. [217] have shown, through computational analyses, the large number of duplication events among tick genes that could be associated with evolutionary pressure through pathogen and/or immune interactions. This would also explain the existence of large multigenic protein families, resulting in redundancy and pluripotency of tick proteins [218]. Despite these challenges, one can imagine that tick proteins with different effector functions might boost the transmission-blocking potential, when used as antigenic combinations. To add to the complexity of tick-host-pathogen interactions, recent insights into the microbiome of ticks show that the microbiome plays an important role in the gut epithelium barrier of ticks and subsequent colonization of the midgut by for instance *Borrelia* [6]. This affects the effectiveness of ticks as a vector for *Borrelia* and possibly also the vectorial capacity for other TBPs. Interestingly, it has recently been shown that *I. scapularis* secretes a protein, PIXR, that modulates the tick gut microbiome and milieu [219]. One might hypothesize that the tick microbiome could also play a role in the effectiveness of, or could be an additional target for, preventive strategies against the tick. Furthermore, adding another layer of complexity, vertebrate host molecules can also interfere with tick-pathogen dynamics. For instance, host IFN-γ acquired through a blood meal from mice infected with *Borrelia* has been shown to induce antimicrobial responses in the tick [220]. This shows that interspecies signaling exists allowing ticks to detect the risk of invading pathogens and mount counter responses. Although the different layers of complexity of tick-host-pathogen interactions show that the development of combined subunit vaccines can be highly challenging, it also reveals the need to search for new and more potent anti-tick vaccine targets. To this end, different consortia have recently been formed [31, 221].

### The opportunities of novel technologies in antigen discovery

Fortunately, tick researchers have more efficient tools available than ever before. The rise of advanced sequencing tools and bioinformatics has increased the power and sensitivity of antigen discovery. The tick genome is amazingly large: for *I. ricinus* the haploid genome size is about 2.65 Gb (for comparison, the human genome is about 3.2 Gb) [222]. This large genome, in combination with dense repetitive regions give transcriptomic analyses an edge over genomic analyses, i.e. for the discovery of relevant vaccine antigens. Different sequencing approaches that have been, or could be, used include RNA sequencing (RNAseq) and Massive Analysis of cDNA Ends (MACE). Both RNAseq and MACE are able to quantify gene expression, but where the strength of RNAseq is the high sequence coverage, the strength of MACE is highly sensitive gene expression quantification. Therefore, these techniques are complementary and when applied together could result in improved transcriptomic analyses. Another technique that has greatly advanced and has helped support these sequencing efforts is proteomic analyses. Sensitive techniques, such as Peptide Mass Fingerprinting by MALDI-MS and shotgun proteomics by precursor ion detection and product ion detection, have been developed enabling the analysis of small quantities of protein that tick researchers are usually left with [223]. Another method that has been used to overcome the challenges in the quantity imposed on tick researchers is the Yeast Surface Display. The use of yeast cells presenting recombinant tick proteins that have post-translational modifications and can be selected and expanded has proven to be a valuable tool in target identification and protein-protein interactions [50, 57, 224].

The design of transcriptomic or proteomic studies for conserved tick proteins involved in TBP transmission is complicated by the variation in transmission times for different TBPs during the tick feeding process. In addition, although genomic studies have been carried out on the salivary/midgut genes of uninfected ticks or tick cell lines, the use of TBP-infected ticks for transcriptomic analyses is still scarce [6], most likely due to the lack of robust models of tick infection for some of these pathogens. An elegant alternative for animal models is the use of artificial membrane feeding systems, which have been the subject of intensive development and refinement as of late [225–228]. These systems could aid the procurement of TBP-infected ticks by allowing them to feed on blood that can be constantly replenished with pathogen cultures. *In vitro* feeding techniques have been established and described for *I. ricinus* and it has been shown for larvae of other tick species that the volume of feeding medium used can go down to less than 1 ml, increasing the suitability of *in vitro* feeding to study tick-pathogen interactions [229, 230]. Another sophisticated technique that has evolved as an invaluable tool in tick vaccine research is RNAi [31]. RNAi exploits the tick’s immune response; double stranded RNA (dsRNA) is injected in the tick, taken up by the cells and cleaved by the RNAse III enzyme DICER. Subsequent small interfering RNA’s are incorporated into the RNA-induced silencing complex, which in turn degrades or inhibits target RNA resulting in gene silencing. In contrast to RNAi in mammals, long (200–300 bp) dsRNA do not result in IFN-induced cell death in ticks and can be used for RNAi, resulting in more efficient knock down of the target gene. Using RNAi and subsequent knockdown, the function of tick genes in tick feeding and/or pathogen transmission can be more rapidly assessed, either *in vitro* or *in vivo.* This could help to narrow the number of candidates that can be further pursued in preclinical relevant settings. The powerful techniques described above give tick researchers the highly needed tools to peel off the complex layers of tick-host-pathogen interactions and to find ways to tip the balance in favor of the host.

### Vaccination awareness

The future development and application of anti-tick vaccines do not only depend on the biological hurdles or technical (im)possibilities. Pharmaceutical companies need to be interested in producing and bringing safe and effective anti-tick vaccines into the market. Clinical phase I/II trials to investigate the safety and immunogenicity in healthy adults, are the first step. Yet, phase III trials assessing the effectiveness of a new vaccine are relatively easy for TBE and LB (i.e. *erythema migrans*), but far more challenging and costly for diseases such as HGA, SFG-rickettsiosis and human babesiosis. Interestingly, a recent cost-effectiveness assessment of a potential anti-tick vaccine protecting against LB and TBEV showed that such a vaccine would be cost-effective in a country where both diseases are endemic, and highlighted which pharmacoeconomic criteria need to be monitored [231]. Given the current health concerns related to LB, a novel vaccine would most likely be highly welcomed by society. On the other hand, the previously commercially available vaccine against LB was taken off the market for various and questionable reasons [232]. Therefore, efforts are needed to address societal prejudices associated with vaccination, including health benefits, risks, and necessity, especially from a public health perspective. Public engagement is important in order to reach the populations at risk, as well as addressing the disparity in the use of these preventive measures in veterinary and human health [177], and both require the involvement of institutions, care providers, researchers, patients and others.

## Conclusions

From the above, it is becoming clear that the control of tick-borne diseases is not only an ‘infectious disease’ problem, but rather a multidisciplinary one. It requires the involvement of geneticists, epidemiologists, immunologists, vector biologists, bioinformaticians, physicians and veterinarians, public health specialists, and the pharmaceutical industry, amongst others. It is also clear that, due to the highly complex multi-angular interactions between microorganisms (pathogens and symbionts), tick vectors and animal or human hosts, the search for the ‘magic bullet’ is not an easy task. But, how far is the goal post exactly? The best way to bite back against tick-borne diseases is to obtain more knowledge on the many aspects of the interaction between ticks, pathogens and mammals and development of tools to study these. As we have described in this review, new powerful tools have enabled substantial progress in the understanding of tick-host-pathogen interactions and the discovery of potential vaccine targets in recent years. Increasing efforts to peel of the complex layers of tick-host-pathogen interactions will provide a higher chance of discovering new and more potent targets for anti-tick vaccines. Therefore, this might be the dawn of a new era where an anti-tick vaccine protecting against the most common TBPs will come to fruition.

## Data Availability

All data generated or analysed during this study are included in this article.

## Abbreviations

TBEV: tick-borne encephalitis virus
LB: Lyme borreliosis
TBE: tick-borne encephalitis
TBPs: tick-borne pathogens
CRASPs: complement regulator-acquiring surface proteins
OspA: outer surface protein
TROSPA: tick receptor for outer surface protein A
TSLPI: tick mannose binding lectin inhibitor
tHRF: tick histamine release factor
Salp15: salivary gland protein of 15 kDa
OspC: outer surface protein C
EAE: experimental autoimmune encephalomyelitis
SAT: saliva-assisted transmission
CCHF: Crimean-Congo haemorrhagic fever
SUB: subolesin
HGA: human granulocytic anaplasmosis
TBF: tick-borne fever
ompA: outer membrane protein A
AipA: infection protein A
IAFGP: *Ixodes* anti-freeze glycoprotein
IAPK1: *Ixodes* p21-activated kinase
SFG: spotted fever group
PTK: phosphotyrosine kinase
FAK: focal adhesion kinase
ABM: actin-based mobility
RNAseq: RNA sequencing
dsRNA: double stranded RNA

## References

[1] Rosenberg Ronald, Lindsey Nicole P., Fischer Marc, Gregory Christopher J., Hinckley Alison F., Mead Paul S., Paz-Bailey Gabriela, Waterman Stephen H., Drexler Naomi A., Kersh Gilbert J., Hooks Holley, Partridge Susanna K., Visser Susanna N., Beard Charles B., Petersen Lyle R. (2018). Vital Signs: Trends in Reported Vectorborne Disease Cases — United States and Territories, 2004–2016. MMWR. Morbidity and Mortality Weekly Report.

[2] Sprong H, Azagi T, Hoornstra D, Nijhof AM, Knorr S, Baarsma ME (2018). Control of Lyme borreliosis and other *Ixodes ricinus*-borne diseases. Parasites Vectors.

[3] European Parliament. Parliament calls for “alarming” spread of Lyme disease to be tackled. 2018. http://www.europarl.europa.eu/news/en/press-room/20181106IPR18328/parliament-calls-for-alarming-spread-of-lyme-disease-to-be-tackled. Accessed 22 Oct 2018.

[4] Paules CI, Marston HD, Bloom ME, Fauci AS (2018). Tickborne diseases—confronting a growing threat. N Engl J Med..

[5] Lehrer AT, Holbrook MR (2011). Tick-borne encephalitis vaccines. J Bioterrorism Biodefense.

[6] de la Fuente J, Antunes S, Bonnet S, Cabezas-Cruz A, Domingos AG, Estrada-Peña A (2017). Tick-pathogen interactions and vector competence: identification of molecular drivers for tick-borne diseases. Front Cell Infect Microbiol..

[7] Vayssier-Taussat M, Kazimirova M, Hubalek Z, Hornok S, Farkas R, Cosson J-F (2015). Emerging horizons for tick-borne pathogens: from the “one pathogen-one disease” vision to the pathobiome paradigm. Future Microbiol.

[8] de la Fuente J, Contreras M, Estrada-Peña A, Cabezas-Cruz A (2017). Targeting a global health problem: vaccine design and challenges for the control of tick-borne diseases. Vaccine..

[9] Fischhoff IR, Keesing F, Ostfeld RS (2017). The tick biocontrol agent *Metarhizium brunneum* (= *M. anisopliae*) (strain F52) does not reduce non-target arthropods. PLoS ONE.

[10] Schorderet-Weber S, Noack S, Selzer PM, Kaminsky R (2017). Blocking transmission of vector-borne diseases. Int J Parasitol Drugs Drug Resist.

[11] de la Fuente J, Contreras M (2015). Tick vaccines: current status and future directions. Expert Rev Vaccines..

[12] Trager W (1939). Acquired immunity to ticks. J Parasitol..

[13] Wikel SK (1996). Host immunity to ticks. Annu Rev Entomol..

[14] Ribeiro JM (1989). Role of saliva in tick/host interactions. Exp Appl Acarol.

[15] Paine SH, Kemp DH, Allen JR (1983). In vitro feeding of *Dermacentor andersoni* (Stiles): effects of histamine and other mediators. Parasitology..

[16] Wada T, Ishiwata K, Koseki H, Ishikura T, Ugajin T, Ohnuma N (2010). Selective ablation of basophils in mice reveals their nonredundant role in acquired immunity against ticks. J Clin Investig.

[17] Anderson JM, Moore IN, Nagata BM, Ribeiro JMC, Valenzuela JG, Sonenshine DE (2017). Ticks, *Ixodes scapularis*, feed repeatedly on white-footed mice despite strong inflammatory response: an expanding paradigm for understanding tick–host interactions. Front Immunol..

[18] Ribeiro JMC, Alarcon-Chaidez F, Francischetti IMB, Mans BJ, Mather TN, Valenzuela JG (2006). An annotated catalog of salivary gland transcripts from *Ixodes scapularis* ticks. Insect Biochem Mol Biol..

[19] Francischetti IMB, Sa-Nunes A, Mans BJ, Santos IM, Ribeiro JMC (2009). The role of saliva in tick feeding. Front Biosci Landmark Ed.

[20] Brossard M, Girardin P (1979). Passive transfer of resistance in rabbits infested with adult *Ixodes ricinus* L: humoral factors influence feeding and egg laying. Experientia.

[21] Roberts JA, Kerr JD (1976). *Boophilus microplus*: passive transfer of resistance in cattle. J Parasitol..

[22] Wikel SK, Allen JR (1976). Acquired resistance to ticks. I. Passive transfer of resistance. Immunology..

[23] Rueckert C, Guzmán CA (2012). Vaccines: from empirical development to rational design. PLoS Pathog..

[24] Carreón D, de la Lastra JMP, Almazán C, Canales M, Ruiz-Fons F, Boadella M (2012). Vaccination with BM86, subolesin and akirin protective antigens for the control of tick infestations in white tailed deer and red deer. Vaccine..

[25] Pal U, Li X, Wang T, Montgomery RR, Ramamoorthi N, Desilva AM (2004). TROSPA, an *Ixodes scapularis* receptor for *Borrelia burgdorferi*. Cell..

[26] de la Fuente J, Almazán C, Canales M, Pérez de la Lastra JM, Kocan KM, Willadsen P (2007). A ten-year review of commercial vaccine performance for control of tick infestations on cattle. Anim Health Res Rev..

[27] Jonsson NN, Matschoss AL, Pepper P, Green PE, Albrecht MS, Hungerford J (2000). Evaluation of tickGARD(PLUS), a novel vaccine against *Boophilus microplus*, in lactating Holstein-Friesian cows. Vet Parasitol..

[28] de la Fuente J, Rodríguez M, Montero C, Redondo M, García-García JC, Méndez L (1999). Vaccination against ticks (*Boophilus* spp.): the experience with the Bm86-based vaccine Gavac. Genet Anal Biomol Eng.

[29] Coumou J, Wagemakers A, Trentelman JJ, Nijhof AM, Hovius JW (2014). Vaccination against Bm86 homologues in rabbits does not impair *Ixodes ricinus* feeding or oviposition. PLoS ONE.

[30] Narasimhan S, Deponte K, Marcantonio N, Liang X, Royce TE, Nelson KF (2007). Immunity against *Ixodes scapularis* salivary proteins expressed within 24 hours of attachment thwarts tick feeding and impairs *Borrelia* transmission. PLoS ONE..

[31] Sprong H, Trentelman J, Seemann I, Grubhoffer L, Rego RO, Hajdušek O (2014). ANTIDotE: anti-tick vaccines to prevent tick-borne diseases in Europe. Parasites Vectors..

[32] Rizzoli A, Hauffe H, Carpi G, Vourc HG, Neteler M, Rosa R (2011). Lyme borreliosis in Europe. Eurosurveillance.

[33] Olson CM, Fikrig E, Anguita J, Rich RR, Fleisher TA, Shearer WT, Schroeder HW, Frew AJ, Weyand CM (2013). Host defenses to spirochetes. Clinical immunology: principles and practice.

[34] Kuehn BM (2013). CDC estimates 300,000 US cases of Lyme disease annually. JAMA.

[35] Hahn MB, Jarnevich CS, Monaghan AJ, Eisen RJ (2016). Modeling the geographic distribution of *Ixodes scapularis* and *Ixodes pacificus* (Acari: Ixodidae) in the Contiguous United States. J Med Entomol.

[36] Hofhuis A, van de Kassteele J, Sprong H, van den Wijngaard CC, Harms MG, Fonville M (2017). Predicting the risk of Lyme borreliosis after a tick bite, using a structural equation model. PLoS ONE.

[37] Medlock JM, Hansford KM, Bormane A, Derdakova M, Estrada-Peña A, George J-C (2013). Driving forces for changes in geographical distribution of *Ixodes ricinus* ticks in Europe. Parasites Vectors.

[38] Rumer L, Sheshukova O, Dautel H, Donoso Mantke O, Niedrig M (2011). Differentiation of medically important Euro-Asian tick species Ixodes ricinus, *Ixodes persulcatus*, *Ixodes hexagonus*, and *Dermacentor reticulatus* by polymerase chain reaction. Vector Borne Zoonotic Dis..

[39] van Duijvendijk G, Coipan C, Wagemakers A, Fonville M, Ersöz J, Oei A (2016). Larvae of *Ixodes ricinus* transmit *Borrelia afzelii* and *B. miyamotoi* to vertebrate hosts. Parasites Vectors..

[40] Strnad M, Hönig V, Růžek D, Grubhoffer L, Rego ROM (2017). Europe-wide meta-analysis of *Borrelia burgdorferi sensu lato* prevalence in questing *Ixodes ricinus* ticks. Appl Environ Microbiol..

[41] Hofmeester TR, Coipan EC, van Wieren SE, Prins HHT, Takken W, Sprong H (2016). Few vertebrate species dominate the *Borrelia burgdorferi s.l.* life cycle. Environ Res Lett..

[42] Dunham-Ems SM, Caimano MJ, Pal U, Wolgemuth CW, Eggers CH, Balic A (2009). Live imaging reveals a biphasic mode of dissemination of *Borrelia burgdorferi* within ticks. J Clin Investig.

[43] Fikrig E, Barthold SW, Kantor FS, Flavell RA (1990). Protection of mice against the Lyme disease agent by immunizing with recombinant OspA. Science..

[44] Zhang L, Zhang Y, Adusumilli S, Liu L, Narasimhan S, Dai J (2011). Molecular interactions that enable movement of the Lyme disease agent from the tick gut into the hemolymph. PLoS Pathog..

[45] Pospisilova T, Urbanova V, Hes O, Kopacek P, Hajdusek O, Sima R (2018). Tracking *Borrelia afzelii* from infected *Ixodes ricinus* nymphs to mice suggests a direct ‘gut-to-mouth’ route of Lyme disease transmission. BioRxiv.

[46] Steere AC, Sikand VK, Meurice F, Parenti DL, Fikrig E, Schoen RT (1998). Vaccination against Lyme disease with recombinant *Borrelia burgdorferi* outer-surface lipoprotein A with adjuvant. N Engl J Med..

[47] Nigrovic LE, Thompson KM (2007). The Lyme vaccine: a cautionary tale. Epidemiol Infect..

[48] Comstedt P, Schüler W, Meinke A, Lundberg U (2017). The novel Lyme borreliosis vaccine VLA15 shows broad protection against *Borrelia* species expressing six different OspA serotypes. PLoS ONE..

[49] Kotsyfakis M, Sá-Nunes A, Francischetti IMB, Mather TN, Andersen JF, Ribeiro JMC (2006). Antiinflammatory and immunosuppressive activity of sialostatin L, a salivary cystatin from the tick *Ixodes scapularis*. J Biol Chem..

[50] Schuijt TJ, Narasimhan S, Daffre S, DePonte K, Hovius JWR, Van’t Veer C (2011). Identification and characterization of *Ixodes scapularis* antigens that elicit tick immunity using yeast surface display. PLoS ONE..

[51] Wagemakers A, Coumou J, Schuijt TJ, Oei A, Nijhof AM, van ’t Veer C (2016). An *Ixodes ricinus* tick salivary lectin pathway inhibitor protects *Borrelia burgdorferi sensu lato* from human complement. Vector Borne Zoonotic Dis..

[52] Schuijt TJ, Bakhtiari K, Daffre S, Deponte K, Wielders SJH, Marquart JA (2013). Factor Xa activation of factor V is of paramount importance in initiating the coagulation system: lessons from a tick salivary protein. Circulation..

[53] Schuijt TJ, Coumou J, Narasimhan S, Dai J, Deponte K, Wouters D (2011). A tick mannose-binding lectin inhibitor interferes with the vertebrate complement cascade to enhance transmission of the lyme disease agent. Cell Host Microbe..

[54] Ramamoorthi N, Narasimhan S, Pal U, Bao F, Yang XF, Fish D (2005). The Lyme disease agent exploits a tick protein to infect the mammalian host. Nature..

[55] Cotté V, Sabatier L, Schnell G, Carmi-Leroy A, Rousselle J-C, Arsène-Ploetze F (2014). Differential expression of *Ixodes ricinus* salivary gland proteins in the presence of the *Borrelia burgdorferi sensu lato* complex. J Proteomics..

[56] Dai J, Narasimhan S, Zhang L, Liu L, Wang P, Fikrig E (2010). Tick histamine release factor is critical for *Ixodes scapularis* engorgement and transmission of the Lyme disease agent. PLoS Pathog..

[57] Narasimhan S, Coumou J, Schuijt TJ, Boder E, Hovius JW, Fikrig E (2014). A tick gut protein with fibronectin III domains aids *Borrelia burgdorferi* congregation to the gut during transmission. PLoS Pathog..

[58] Coumou J, Narasimhan S, Trentelman JJ, Wagemakers A, Koetsveld J, Ersoz JI (2016). *Ixodes scapularis* dystroglycan-like protein promotes *Borrelia burgdorferi* migration from the gut. J Mol Med.

[59] Embers ME, Narasimhan S (2013). Vaccination against Lyme disease: past, present, and future. Front Cell Infect Microbiol..

[60] Hovius JWR, van Dam AP, Fikrig E (2007). Tick–host–pathogen interactions in Lyme borreliosis. Trends Parasitol..

[61] Klouwens MJ, Trentelman JJ, Hovius JWR. Anti-tick vaccines to prevent tick-borne diseases: an overview and a glance at the future. In: Ecol Prev Lyme Borreliosis. Wageningen: Wageningen Academic Publishers; 2016. p. 295–316.

[62] Merino O, Alberdi P, Pérez de la Lastra JM, de la Fuente J (2013). Tick vaccines and the control of tick-borne pathogens. Front Cell Infect Microbiol..

[63] Brossard M, Fivaz V (1982). *Ixodes ricinus* L.: mast cells, basophils and eosinophils in the sequence of cellular events in the skin of infested or re-infested rabbits. Parasitology..

[64] Monteiro GER, Bechara GH (2008). Cutaneous basophilia in the resistance of goats to *Amblyomma cajennense* nymphs after repeated infestations. Ann N Y Acad Sci..

[65] Kemp DH, Bourne A (1980). *Boophilus microplus*: the effect of histamine on the attachment of cattle-tick larvae—studies *in vivo* and *in vitro*. Parasitology..

[66] Das S, Banerjee G, DePonte K, Marcantonio N, Kantor FS, Fikrig E (2001). Salp25D, an *Ixodes scapularis* antioxidant, is 1 of 14 immunodominant antigens in engorged tick salivary glands. J Infect Dis..

[67] Dai J, Wang P, Adusumilli S, Booth CJ, Narasimhan S, Anguita J (2009). Antibodies against a tick protein, Salp15, protect mice from the Lyme disease agent. Cell Host Microbe..

[68] Anguita J, Ramamoorthi N, Hovius JWR, Das S, Thomas V, Persinski R (2002). Salp15, an ixodes scapularis salivary protein, inhibits CD4(+) T cell activation. Immunity..

[69] Garg R, Juncadella IJ, Ramamoorthi N, Ananthanarayanan SK, Thomas V (1950). Cutting edge: CD4 is the receptor for the tick saliva immunosuppressor, Salp15. J Immunol..

[70] Juncadella IJ, Garg R, Boone CD, Anguita J, Krueger JK (2008). Conformational rearrangement within the soluble domains of the CD4 receptor is ligand-specific. J Biol Chem..

[71] Juncadella IJ, Garg R, Ananthnarayanan SK, Yengo CM, Anguita J (2007). T-cell signaling pathways inhibited by the tick saliva immunosuppressor, Salp15. FEMS Immunol Med Microbiol..

[72] Hovius JWR, de Jong MAWP, den Dunnen J, Litjens M, Fikrig E, van der Poll T (2008). Salp15 binding to DC-SIGN inhibits cytokine expression by impairing both nucleosome remodeling and mRNA stabilization. PLoS Pathog..

[73] Schuijt TJ, Hovius JWR, van Burgel ND, Ramamoorthi N, Fikrig E, van Dam AP (2008). The tick salivary protein Salp15 inhibits the killing of serum-sensitive *Borrelia burgdorferi sensu lato* isolates. Infect Immun..

[74] Hovius JWR, Levi M, Fikrig E (2008). Salivating for knowledge: potential pharmacological agents in tick saliva. PLoS Med..

[75] Paveglio SA, Allard J, Mayette J, Whittaker LA, Juncadella I, Anguita J (2007). The tick salivary protein, Salp15, inhibits the development of experimental asthma. J Immunol..

[76] Juncadella IJ, Bates TC, Suleiman R, Monteagudo-Mera A, Olson CM, Navasa N (2010). The tick saliva immunosuppressor, Salp15, contributes to Th17-induced pathology during experimental autoimmune encephalomyelitis. Biochem Biophys Res Commun..

[77] Süss J (2011). Tick-borne encephalitis 2010: epidemiology, risk areas, and virus strains in Europe and Asia—an overview. Ticks Tick Borne Dis.

[78] Donoso Mantke O, Escadafal C, Niedrig M, Pfeffer M, Working Group For Tick-Borne Encephalitis Virus C (2011). Tick-borne encephalitis in Europe, 2007 to 2009. Eurosurveillance..

[79] Dobler G (2010). Zoonotic tick-borne flaviviruses. Vet Microbiol..

[80] Amicizia D, Domnich A, Panatto D, Lai PL, Cristina ML, Avio U (2013). Epidemiology of tick-borne encephalitis (TBE) in Europe and its prevention by available vaccines. Hum Vaccines Immunother..

[81] Gritsun TS, Lashkevich VA, Gould EA (2003). Tick-borne encephalitis. Antiviral Res..

[82] Šmit R, Postma MJ (2016). Vaccines for tick-borne diseases and cost-effectiveness of vaccination: a public health challenge to reduce the diseases’ burden. Expert Rev Vaccines..

[83] Nuttall PA, Labuda M (2003). Dynamics of infection in tick vectors and at the tick–host interface. Adv Virus Res..

[84] Randolph SE (2004). Tick ecology: processes and patterns behind the epidemiological risk posed by ixodid ticks as vectors. Parasitology..

[85] Slovák M, Kazimírová M, Siebenstichová M, Ustaníková K, Klempa B, Gritsun T (2014). Survival dynamics of tick-borne encephalitis virus in *Ixodes ricinus* ticks. Ticks Tick Borne Dis..

[86] Labuda M, Danielova V, Jones LD, Nuttall PA (1993). Amplification of tick-borne encephalitis virus infection during co-feeding of ticks. Med Vet Entomol..

[87] Labuda M, Kozuch O, Zuffová E, Elecková E, Hails RS, Nuttall PA (1997). Tick-borne encephalitis virus transmission between ticks cofeeding on specific immune natural rodent hosts. Virology..

[88] Labuda M, Austyn JM, Zuffova E, Kozuch O, Fuchsberger N, Lysy J (1996). Importance of localized skin infection in tick-borne encephalitis virus transmission. Virology..

[89] Nuttall PA, Labuda M, Bowman AS, Nuttall PA (2008). Saliva-assisted transmission of tick-borne pathogens. Ticks: biology, disease and control.

[90] Kazimírová M, Thangamani S, Bartíková P, Hermance M, Holíková V, Štibrániová I (2017). Tick-borne viruses and biological processes at the tick–host–virus interface. Front Cell Infect Microbiol..

[91] Šimo L, Kazimirova M, Richardson J, Bonnet SI (2017). The essential role of tick salivary glands and saliva in tick feeding and pathogen transmission. Front Cell Infect Microbiol..

[92] Kotsyfakis M, Karim S, Andersen JF, Mather TN, Ribeiro JMC (2007). Selective cysteine protease inhibition contributes to blood-feeding success of the tick *Ixodes scapularis*. J Biol Chem..

[93] Lieskovská J, Páleníková J, Širmarová J, Elsterová J, Kotsyfakis M, Campos Chagas A (2015). Tick salivary cystatin sialostatin L2 suppresses IFN responses in mouse dendritic cells. Parasite Immunol..

[94] Manjunathachar HV, Kumar B, Saravanan BC, Choudhary S, Mohanty AK, Nagar G (2019). Identification and characterization of vaccine candidates against *Hyalomma anatolicum*—vector of Crimean-Congo haemorrhagic fever virus. Transbound Emerg Dis..

[95] Trimnell AR, Davies GM, Lissina O, Hails RS, Nuttall PA (2005). A cross-reactive tick cement antigen is a candidate broad-spectrum tick vaccine. Vaccine..

[96] Trimnell AR, Hails RS, Nuttall PA (2002). Dual action ectoparasite vaccine targeting “exposed” and “concealed” antigens. Vaccine..

[97] Labuda M, Trimnell AR, Licková M, Kazimírová M, Davies GM, Lissina O (2006). An antivector vaccine protects against a lethal vector-borne pathogen. PLoS Pathog..

[98] Zivkovic Z, Torina A, Mitra R, Alongi A, Scimeca S, Kocan KM (2010). Subolesin expression in response to pathogen infection in ticks. BMC Immunol..

[99] Galindo RC, Doncel-Pérez E, Zivkovic Z, Naranjo V, Gortazar C, Mangold AJ (2009). Tick subolesin is an ortholog of the akirins described in insects and vertebrates. Dev Comp Immunol..

[100] Mangold AJ, Galindo RC, de la Fuente J, Response to the commentary of D (2009). Macqueen on: Galindo RC, Doncel-Pérez E, Zivkovic Z, Naranjo V, Gortazar C, Mangold AJ, et al. Tick subolesin is an ortholog of the akirins described in insects and vertebrates [Dev. Comp. Immunol. 33 (2009) 612–617]. Dev Comp Immunol.

[101] de la Fuente J, Almazán C, Blouin EF, Naranjo V, Kocan KM (2006). Reduction of tick infections with *Anaplasma marginale* and *A. phagocytophilum* by targeting the tick protective antigen subolesin. Parasitol Res..

[102] Bensaci M, Bhattacharya D, Clark R, Hu LT (2012). Oral vaccination with vaccinia virus expressing the tick antigen subolesin inhibits tick feeding and transmission of *Borrelia burgdorferi*. Vaccine..

[103] Merino O, Almazán C, Canales M, Villar M, Moreno-Cid JA, Galindo RC (2011). Targeting the tick protective antigen subolesin reduces vector infestations and pathogen infection *by Anaplasma marginale* and *Babesia bigemina*. Vaccine..

[104] Havlíková S, Ličková M, Ayllón N, Roller L, Kazimírová M, Slovák M (2013). Immunization with recombinant subolesin does not reduce tick infection with tick-borne encephalitis virus nor protect mice against disease. Vaccine..

[105] Stuen S, Granquist EG, Silaghi C (2013). *Anaplasma phagocytophilum*—a widespread multi-host pathogen with highly adaptive strategies. Front Cell Infect Microbiol..

[106] Gordon WS, Brownlee A, Wilson DR, Macleod J (1932). Tick-borne fever: a hitherto undescribed disease of sheep. J Comp Pathol Ther..

[107] Chen SM, Dumler JS, Bakken JS, Walker DH (1994). Identification of a granulocytotropic *Ehrlichia* species as the etiologic agent of human disease. J Clin Microbiol..

[108] Woldehiwet Z (2010). The natural history of *Anaplasma phagocytophilum*. Vet Parasitol..

[109] Bakken JS, Dumler JS (2015). Human granulocytic anaplasmosis. Infect Dis Clin N Am..

[110] de la Fuente J, Estrada-Peña A, Cabezas-Cruz A, Kocan KM (2016). *Anaplasma phagocytophilum* uses common strategies for infection of ticks and vertebrate hosts. Trends Microbiol..

[111] Telford SR, Dawson JE, Katavolos P, Warner CK, Kolbert CP, Persing DH (1996). Perpetuation of the agent of human granulocytic ehrlichiosis in a deer tick-rodent cycle. Proc Natl Acad Sci USA.

[112] Stuen S, Okstad W, Artursson K, Al-Khedery B, Barbet A, Granquist EG (2015). Lambs immunized with an inactivated variant of *Anaplasma phagocytophilum*. Acta Vet Scand..

[113] Ojogun N, Kahlon A, Ragland SA, Troese MJ, Mastronunzio JE, Walker NJ (2012). *Anaplasma phagocytophilum* outer membrane protein A interacts with sialylated glycoproteins to promote infection of mammalian host cells. Infect Immun..

[114] Kahlon A, Ojogun N, Ragland SA, Seidman D, Troese MJ, Ottens AK (2013). *Anaplasma phagocytophilum* Asp14 is an invasin that interacts with mammalian host cells via its C terminus to facilitate infection. Infect Immun..

[115] Seidman D, Ojogun N, Walker NJ, Mastronunzio J, Kahlon A, Hebert KS (2014). *Anaplasma phagocytophilum* surface protein AipA mediates invasion of mammalian host cells. Cell Microbiol..

[116] Seidman D, Hebert KS, Truchan HK, Miller DP, Tegels BK, Marconi RT (2015). Essential domains of *Anaplasma phagocytophilum* invasins utilized to infect mammalian host cells. PLoS Pathog..

[117] Contreras M, Alberdi P, Mateos-Hernández L, Fernández de Mera IG, García-Pérez AL, Vancová M (2017). *Anaplasma phagocytophilum* MSP4 and HSP70 proteins are involved in interactions with host cells during pathogen infection. Front Cell Infect Microbiol..

[118] Sukumaran B, Narasimhan S, Anderson JF, DePonte K, Marcantonio N, Krishnan MN (2006). An *Ixodes scapularis* protein required for survival of *Anaplasma phagocytophilum* in tick salivary glands. J Exp Med..

[119] Liu L, Narasimhan S, Dai J, Zhang L, Cheng G, Fikrig E (2011). *Ixodes scapularis* salivary gland protein P11 facilitates migration of *Anaplasma phagocytophilum* from the tick gut to salivary glands. EMBO Rep..

[120] Ayllón N, Villar M, Galindo RC, Kocan KM, Šíma R, López JA (2015). Systems biology of tissue-specific response to *Anaplasma phagocytophilum* reveals differentiated apoptosis in the tick vector *Ixodes scapularis*. PLoS Genet..

[121] Villar M, Ayllón N, Alberdi P, Moreno A, Moreno M, Tobes R (2015). Integrated metabolomics, transcriptomics and proteomics identifies metabolic pathways affected by *Anaplasma phagocytophilum* infection in tick cells. Mol Cell Proteomics MCP..

[122] Neelakanta G, Sultana H, Fish D, Anderson JF, Fikrig E (2010). *Anaplasma phagocytophilum* induces *Ixodes scapularis* ticks to express an antifreeze glycoprotein gene that enhances their survival in the cold. J Clin Investig.

[123] Pedra JHF, Narasimhan S, Rendić D, DePonte K, Bell-Sakyi L, Wilson IBH (2010). Fucosylation enhances colonization of ticks by *Anaplasma phagocytophilum*. Cell Microbiol..

[124] Contreras M, Alberdi P, Fernández De Mera IG, Krull C, Nijhof A, Villar M (2017). Vaccinomics approach to the identification of candidate protective antigens for the control of tick vector infestations and *Anaplasma phagocytophilum* infection. Front Cell Infect Microbiol..

[125] Sultana H, Neelakanta G, Kantor FS, Malawista SE, Fish D, Montgomery RR (2010). *Anaplasma phagocytophilum* induces actin phosphorylation to selectively regulate gene transcription in *Ixodes scapularis* ticks. J Exp Med..

[126] Heisig M, Abraham NM, Liu L, Neelakanta G, Mattessich S, Sultana H (2014). Antivirulence properties of an antifreeze protein. Cell Rep..

[127] Abraham NM, Liu L, Jutras BL, Yadav AK, Narasimhan S, Gopalakrishnan V (2017). Pathogen-mediated manipulation of arthropod microbiota to promote infection. Proc Natl Acad Sci USA.

[128] de la Fuente J, Canales M, Kocan KM (2006). The importance of protein glycosylation in development of novel tick vaccine strategies. Parasite Immunol..

[129] de la Fuente J, Moreno-Cid JA, Galindo RC, Almazan C, Kocan KM, Merino O (2013). Subolesin/Akirin vaccines for the control of arthropod vectors and vectorborne pathogens. Transbound Emerg Dis..

[130] Portillo A, Santibáñez S, García-Álvarez L, Palomar AM, Oteo JA (2015). Rickettsioses in Europe. Microbes Infect..

[131] Eremeeva ME, Dasch GA (2015). Challenges posed by tick-borne rickettsiae: eco-epidemiology and public health implications. Front Public Health..

[132] Sahni SK, Narra HP, Sahni A, Walker DH (2013). Recent molecular insights into rickettsial pathogenesis and immunity. Future Microbiol..

[133] Botelho-Nevers E, Socolovschi C, Raoult D, Parola P (2012). Treatment of *Rickettsia* spp. infections: a review. Expert Rev Anti Infect Ther..

[134] Chan YG-Y, Riley SP, Martinez JJ (2010). Adherence to and invasion of host cells by spotted fever group *Rickettsia* species. Front Microbiol..

[135] Walker DH (2009). The realities of biodefense vaccines against *Rickettsia*. Vaccine..

[136] Richards AL (2004). Rickettsial vaccines: the old and the new. Expert Rev Vaccines..

[137] El Karkouri K, Kowalczewska M, Armstrong N, Azza S, Fournier P-E, Raoult D (2017). Multi-omics analysis sheds light on the evolution and the intracellular lifestyle strategies of spotted fever group *Rickettsia* spp. Front Microbiol..

[138] Walker DH, Ismail N (2008). Emerging and re-emerging rickettsioses: endothelial cell infection and early disease events. Nat Rev Microbiol..

[139] Ireton K (2013). Molecular mechanisms of cell–cell spread of intracellular bacterial pathogens. Open Biol..

[140] Kuehl CJ, Dragoi A-M, Talman A, Agaisse H (2015). Bacterial spread from cell to cell: beyond actin-based motility. Trends Microbiol..

[141] Goldberg MB (2001). Actin-based motility of intracellular microbial pathogens. Microbiol Mol Biol Rev..

[142] Petchampai N, Sunyakumthorn P, Banajee KH, Verhoeve VI, Kearney MT, Macaluso KR (2015). Identification of host proteins involved in rickettsial invasion of tick cells. Infect Immun..

[143] Martinez JJ, Cossart P (2004). Early signaling events involved in the entry of *Rickettsia conorii* into mammalian cells. J Cell Sci..

[144] Petchampai N, Sunyakumthorn P, Guillotte ML, Verhoeve VI, Banajee KH, Kearney MT (2014). Novel identification of *Dermacentor variabilis* Arp2/3 complex and its role in rickettsial infection of the arthropod vector. PLoS ONE..

[145] Jeng RL, Goley ED, D’Alessio JA, Chaga OY, Svitkina TM, Borisy GG (2004). A *Rickettsia* WASP-like protein activates the Arp2/3 complex and mediates actin-based motility. Cell Microbiol..

[146] Oliver JD, Burkhardt NY, Felsheim RF, Kurtti TJ, Munderloh UG (2014). Motility characteristics are altered for *Rickettsia bellii* transformed to overexpress a heterologous rickA gene. Appl Environ Microbiol..

[147] Petchampai N, Sunyakumthorn P, Guillotte ML, Thepparit C, Kearney MT, Mulenga A (2014). Molecular and functional characterization of vacuolar-ATPase from the American dog tick *Dermacentor variabilis*. Insect Mol Biol..

[148] Charpentier BM, Bach MA, Lang P, Martin B, Fries D (1987). Phenotypic composition and *in vitro* functional capacities of unmodified fresh cells infiltrating acutely rejected human kidney allografts. Transplantation..

[149] Macaluso KR, Mulenga A, Simser JA, Azad AF (2003). Differential expression of genes in uninfected and rickettsia-infected *Dermacentor variabilis* ticks as assessed by differential-display PCR. Infect Immun..

[150] Sunyakumthorn P, Petchampai N, Grasperge BJ, Kearney MT, Sonenshine DE, Macaluso KR (2013). Gene expression of tissue-specific molecules in *ex vivo Dermacentor variabilis* (Acari: Ixodidae) during rickettsial exposure. J Med Entomol..

[151] Mulenga A, Simser JA, Macaluso KR, Azad AF (2004). Stress and transcriptional regulation of tick ferritin HC. Insect Mol Biol..

[152] Mulenga A, Macaluso KR, Simser JA, Azad AF (2003). Dynamics of *Rickettsia*-tick interactions: identification and characterization of differentially expressed mRNAs in uninfected and infected *Dermacentor variabilis*. Insect Mol Biol..

[153] Rizzoli A, Silaghi C, Obiegala A, Rudolf I, Hubálek Z, Földvári G (2014). *Ixodes ricinus* and its transmitted pathogens in urban and peri-urban areas in Europe: new hazards and relevance for public health. Front Public Health..

[154] Speck S, Kern T, Aistleitner K, Dilcher M, Dobler G, Essbauer S (2018). *In vitro* studies of *Rickettsia*-host cell interactions: confocal laser scanning microscopy of *Rickettsia helvetica*-infected eukaryotic cell lines. PLoS Negl Trop Dis..

[155] Elfving K, Lukinius A, Nilsson K (2012). Life cycle, growth characteristics and host cell response of *Rickettsia helvetica* in a Vero cell line. Exp Appl Acarol..

[156] Socolovschi C, Mediannikov O, Raoult D, Parola P (2009). The relationship between spotted fever group rickettsiae and ixodid ticks. Vet Res..

[157] Ahantarig A, Trinachartvanit W, Baimai V, Grubhoffer L (2013). Hard ticks and their bacterial endosymbionts (or would be pathogens). Folia Microbiol.

[158] Narasimhan S, Fikrig E (2015). Tick microbiome: the force within. Trends Parasitol..

[159] Bonnet SI, Binetruy F, Hernández-Jarguín AM, Duron O (2017). The tick microbiome: why non-pathogenic microorganisms matter in tick biology and pathogen transmission. Front Cell Infect Microbiol..

[160] Homer MJ, Aguilar-Delfin I, Telford SR, Krause PJ, Persing DH (2000). Babesiosis. Clin Microbiol Rev..

[161] Hunfeld K-P, Hildebrandt A, Gray JS (2008). Babesiosis: recent insights into an ancient disease. Int J Parasitol..

[162] Zintl A, Mulcahy G, Skerrett HE, Taylor SM, Gray JS (2003). *Babesia divergens*, a bovine blood parasite of veterinary and zoonotic importance. Clin Microbiol Rev..

[163] Leiby DA (2011). Transfusion-transmitted *Babesia* spp.: bull’s-eye on *Babesia microti*. Clin Microbiol Rev..

[164] Kjemtrup AM, Conrad PA (2000). Human babesiosis: an emerging tick-borne disease. Int J Parasitol..

[165] Gohil S, Herrmann S, Günther S, Cooke BM (2013). Bovine babesiosis in the 21st century: advances in biology and functional genomics. Int J Parasitol..

[166] Bock R, Jackson L, de Vos A, Jorgensen W (2004). Babesiosis of cattle. Parasitology..

[167] Wise LN, Pelzel-McCluskey AM, Mealey RH, Knowles DP (2014). Equine piroplasmosis. Vet Clin N Am Equine Pract.

[168] Irwin PJ (2009). Canine babesiosis: from molecular taxonomy to control. Parasites Vectors..

[169] Florin-Christensen M, Suarez CE, Rodriguez AE, Flores DA, Schnittger L (2014). Vaccines against bovine babesiosis: where we are now and possible roads ahead. Parasitology.

[170] Suarez CE, Noh S (2011). Emerging perspectives in the research of bovine babesiosis and anaplasmosis. Vet Parasitol..

[171] Vannier E, Krause PJ (2012). Human babesiosis. N Engl J Med..

[172] Häselbarth K, Tenter AM, Brade V, Krieger G, Hunfeld K-P (2007). First case of human babesiosis in Germany—clinical presentation and molecular characterisation of the pathogen. Int J Med Microbiol IJMM..

[173] Herwaldt BL, Cacciò S, Gherlinzoni F, Aspöck H, Slemenda SB, Piccaluga P (2003). Molecular characterization of a non-*Babesia divergens* organism causing zoonotic babesiosis in Europe. Emerg Infect Dis..

[174] Conrad PA, Kjemtrup AM, Carreno RA, Thomford J, Wainwright K, Eberhard M (2006). Description of *Babesia duncani* n.csp. (Apicomplexa: Babesiidae) from humans and its differentiation from other piroplasms. Int J Parasitol..

[175] Bloch EM, Herwaldt BL, Leiby DA, Shaieb A, Herron RM, Chervenak M (2012). The third described case of transfusion-transmitted *Babesia duncani*. Transfusion.

[176] Lobo CA, Cursino-Santos JR, Alhassan A, Rodrigues M (2013). *Babesia*: an emerging infectious threat in transfusion medicine. PLoS Pathog..

[177] Vannier E, Gewurz BE, Krause PJ (2008). Human babesiosis. Infect Dis Clin N Am..

[178] Krause PJ, Lepore T, Sikand VK, Gadbaw J, Burke G, Telford SR (2000). Atovaquone and azithromycin for the treatment of babesiosis. N Engl J Med..

[179] Vannier E, Krause PJ (2009). Update on babesiosis. Interdiscip Perspect Infect Dis..

[180] Krause PJ, Gewurz BE, Hill D, Marty FM, Vannier E, Foppa IM (2008). Persistent and relapsing babesiosis in immunocompromised patients. Clin Infect Dis..

[181] Krause PJ (2002). Babesiosis. Med Clin N Am..

[182] Rosner F, Zarrabi MH, Benach JL, Habicht GS (1984). Babesiosis in splenectomized adults. Review of 22 reported cases. Am J Med..

[183] Stowell CP, Gelfand JA, Shepard J-AO, Kratz A (2007). Case records of the Massachusetts General Hospital. Case 17–2007. A 25-year-old woman with relapsing fevers and recent onset of dyspnea. N Engl J Med..

[184] Rodriguez AE, Florin-Christensen M, Flores DA, Echaide I, Suarez CE, Schnittger L (2014). The glycosylphosphatidylinositol-anchored protein repertoire of *Babesia bovis* and its significance for erythrocyte invasion. Ticks Tick Borne Dis..

[185] Kleuskens J, Moubri-Menage K, Rohwer A, Schetters TPM. Canine babesiosis vaccine antigen. 2012. https://patents.google.com/patent/WO2012089748A1/en. Accessed 22 Oct 2018.

[186] Yabsley MJ, Shock BC (2013). Natural history of zoonotic *Babesia*: role of wildlife reservoirs. Int J Parasitol Parasites Wildl..

[187] Vannier EG, Diuk-Wasser MA, Ben Mamoun C, Krause PJ (2015). Babesiosis. Infect Dis Clin N Am..

[188] Gray J, Zintl A, Hildebrandt A, Hunfeld K-P, Weiss L (2010). Zoonotic babesiosis: overview of the disease and novel aspects of pathogen identity. Ticks Tick Borne Dis..

[189] Benezra D, Brown AE, Polsky B, Gold JW, Armstrong D (1987). Babesiosis and infection with human immunodeficiency virus (HIV). Ann Intern Med..

[190] Falagas ME, Klempner MS (1996). Babesiosis in patients with AIDS: a chronic infection presenting as fever of unknown origin. Clin Infect Dis Off Publ Infect Dis Soc Am..

[191] Froberg MK, Dannen D, Bakken JS (2004). Babesiosis and HIV. Lancet Lond Engl.

[192] Krause PJ, Telford SR, Spielman A, Sikand V, Ryan R, Christianson D (1996). Concurrent Lyme disease and babesiosis. Evidence for increased severity and duration of illness. JAMA..

[193] Krause PJ, McKay K, Thompson CA, Sikand VK, Lentz R, Lepore T (2002). Disease-specific diagnosis of coinfecting tickborne zoonoses: babesiosis, human granulocytic ehrlichiosis, and Lyme disease. Clin Infect Dis..

[194] Sonenshine DE, Roe RM (2014). Biology of ticks.

[195] Becker CAM, Bouju-Albert A, Jouglin M, Chauvin A, Malandrin L (2009). Natural transmission of zoonotic Babesia spp. by *Ixodes ricinus* ticks. Emerg Infect Dis..

[196] Bonnet S, Jouglin M, L’Hostis M, Chauvin A (2007). *Babesia* sp. EU1 from roe deer and transmission within *Ixodes ricinus*. Emerg Infect Dis..

[197] Bonnet S, Brisseau N, Hermouet A, Jouglin M, Chauvin A (2009). Experimental *in vitro* transmission of *Babesia* sp. (EU1) by *Ixodes ricinus*. Vet Res..

[198] Cieniuch S, Stańczak J, Ruczaj A (2009). The first detection of *Babesia* EU1 and *Babesia canis canis* in *Ixodes ricinus* ticks (Acari, Ixodidae) collected in urban and rural areas in northern Poland. Pol J Microbiol..

[199] Zintl A, Finnerty EJ, Murphy TM, de Waal T, Gray JS (2011). Babesias of red deer (*Cervus elaphus*) in Ireland. Vet Res..

[200] Nikol’skii SN, Pozov SA (1972). *Ixodes ricinus* ticks as carriers of *Babesia capreoli* in the roe deer. Veterinariia..

[201] Gray J, von Stedingk LV, Gürtelschmid M, Granström M (2002). Transmission studies of *Babesia microti* in *Ixodes ricinus* ticks and gerbils. J Clin Microbiol..

[202] Rudzinska MA, Spielman A, Riek RF, Lewengrub SJ, Piesman J (1979). Intraerythrocytic, “gametocytes” of Babesia microti and their maturation in ticks. Can J Zool..

[203] Mehlhorn H, Shein E (1984). The piroplasms: life cycle and sexual stages. Adv Parasitol..

[204] Piesman J, Karakashian SJ, Lewengrub S, Rudzinska MA, Spielmank A (1986). Development of *Babesia microti* sporozoites in adult *Ixodes dammini*. Int J Parasitol..

[205] Karakashian SJ, Rudzinska MA, Spielman A, Lewengrub S, Piesman J, Shoukrey N (1983). Ultrastructural studies on sporogony of *Babesia microti* in salivary gland cells of the tick *Ixodes dammini*. Cell Tissue Res..

[206] Cen-Aguilar JF, Rodríguez-Vivas RI, Domínguez-Alpizar JL, Wagner GG (1998). Studies on the effect of infection by *Babesia* sp. on oviposition of *Boophilus microplus* engorged females naturally infected in the Mexican tropics. Vet Parasitol..

[207] Hajdušek O, Síma R, Ayllón N, Jalovecká M, Perner J, de la Fuente J (2013). Interaction of the tick immune system with transmitted pathogens. Front Cell Infect Microbiol..

[208] Tsuji N, Fujisaki K (2007). Longicin plays a crucial role in inhibiting the transmission of *Babesia* parasites in the vector tick *Haemaphysalis longicornis*. Future Microbiol..

[209] Tsuji N, Battsetseg B, Boldbaatar D, Miyoshi T, Xuan X, Oliver JH (2007). Babesial vector tick defensin against *Babesia* sp. parasites. Infect Immun..

[210] Tsuji N, Miyoshi T, Battsetseg B, Matsuo T, Xuan X, Fujisaki K (2008). A cysteine protease is critical for *Babesia* spp. transmission in *Haemaphysalis* ticks. PLoS Pathog..

[211] Zhu J, Yin R, Wu H, Yi J, Luo L, Dong G (2006). Cystatin C as a reliable marker of renal function following heart valve replacement surgery with cardiopulmonary bypass. Clin Chim Acta Int J Clin Chem..

[212] Boldbaatar D, Battsetseg B, Matsuo T, Hatta T, Umemiya-Shirafuji R, Xuan X (2008). Tick vitellogenin receptor reveals critical role in oocyte development and transovarial transmission of *Babesia* parasite. Biochem Cell Biol Biochim Biol Cell..

[213] Antunes S, Galindo RC, Almazán C, Rudenko N, Golovchenko M, Grubhoffer L (2012). Functional genomics studies of *Rhipicephalus* (*Boophilus*) *annulatus* ticks in response to infection with the cattle protozoan parasite, *Babesia bigemina*. Int J Parasitol..

[214] Rachinsky A, Guerrero FD, Scoles GA (2007). Differential protein expression in ovaries of uninfected and *Babesia*-infected southern cattle ticks, *Rhipicephalus* (*Boophilus*) *microplus*. Insect Biochem Mol Biol..

[215] Rachinsky A, Guerrero FD, Scoles GA (2008). Proteomic profiling of *Rhipicephalus* (*Boophilus*) *microplus* midgut responses to infection with *Babesia bovis*. Vet Parasitol..

[216] Heekin AM, Guerrero FD, Bendele KG, Saldivar L, Scoles GA, Gondro C (2012). Analysis of *Babesia bovis* infection-induced gene expression changes in larvae from the cattle tick, *Rhipicephalus* (*Boophilus*) *microplus*. Parasites Vectors..

[217] Van Zee JP, Schlueter JA, Schlueter S, Dixon P, Sierra CAB, Hill CA (2016). Paralog analyses reveal gene duplication events and genes under positive selection in *Ixodes scapularis* and other ixodid ticks. BMC Genomics..

[218] Chmelař J, Kotál J, Kopecky J, Pedra JH, Kotsyfakis M (2016). All for one and one for all on the tick-host battlefield. Trends Parasitol..

[219] Narasimhan S, Schuijt TJ, Abraham NM, Rajeevan N, Coumou J, Graham M (2017). Modulation of the tick gut milieu by a secreted tick protein favors *Borrelia burgdorferi* colonization. Nat Commun..

[220] Smith AA, Navasa N, Yang X, Wilder CN, Buyuktanir O, Marques A (2016). Cross-species interferon signaling boosts microbicidal activity within the tick vector. Cell Host Microbe..

[221] Schetters T, Bishop R, Crampton M, Kopáček P, Lew-Tabor A, Maritz-Olivier C (2016). Cattle tick vaccine researchers join forces in CATVAC. Parasites Vectors..

[222] Cramaro WJ, Hunewald OE, Bell-Sakyi L, Muller CP (2017). Genome scaffolding and annotation for the pathogen vector *Ixodes ricinus* by ultra-long single molecule sequencing. Parasites Vectors..

[223] Iwamoto N, Shimada T (2018). Recent advances in mass spectrometry-based approaches for proteomics and biologics: great contribution for developing therapeutic antibodies. Pharmacol Ther..

[224] Chao G, Lau WL, Hackel BJ, Sazinsky SL, Lippow SM, Wittrup KD (2006). Isolating and engineering human antibodies using yeast surface display. Nat Protoc..

[225] Maeda H, Hatta T, Alim MA, Tsubokawa D, Mikami F, Matsubayashi M (2016). Establishment of a novel tick-*Babesia* experimental infection model. Sci Rep..

[226] Krull C, Böhme B, Clausen P-H, Nijhof AM (2017). Optimization of an artificial tick feeding assay for *Dermacentor reticulatus*. Parasites Vectors..

[227] Böhme B, Krull C, Clausen P-H, Nijhof AM (2018). Evaluation of a semi-automated in vitro feeding system for *Dermacentor reticulatus* and *Ixodes ricinus* adults. Parasitol Res..

[228] Romano D, Stefanini C, Canale A, Benelli G (2018). Artificial blood feeders for mosquito and ticks—where from, where to?. Acta Trop..

[229] Kröber T, Guerin PM (2007). *In vitro* feeding assays for hard ticks. Trends Parasitol..

[230] Trentelman JJA, Kleuskens JAGM, van de Crommert J, Schetters TPM (2017). A new method for in vitro feeding of *Rhipicephalus australis* (formerly *Rhipicephalus microplus*) larvae: a valuable tool for tick vaccine development. Parasites Vectors..

[231] Mihajlović J, Hovius JWR, Sprong H, Bogovič P, Postma MJ, Strle F (2019). Cost-effectiveness of a potential anti-tick vaccine with combined protection against Lyme borreliosis and tick-borne encephalitis in Slovenia. Ticks Tick Borne Dis..

[232] Plotkin SA (2016). Need for a new Lyme disease vaccine. N Engl J Med..

